# Out-of-Sequence Signal 3 as a Mechanism for Virus-Induced Immune Suppression of CD8 T Cell Responses

**DOI:** 10.1371/journal.ppat.1004357

**Published:** 2014-09-25

**Authors:** Stina L. Urban, Raymond M. Welsh

**Affiliations:** Department of Pathology, Immunology and Virology Program, University of Massachusetts Medical School, Worcester, Massachusetts, United States of America; Nationwide Children's Hospital, United States of America

## Abstract

Virus infections are known to induce a transient state of immune suppression often associated with an inhibition of T cell proliferation in response to mitogen or cognate-antigen stimulation. Recently, virus-induced immune suppression has been linked to responses to type 1 interferon (IFN), a signal 3 cytokine that normally can augment the proliferation and differentiation of T cells exposed to antigen (signal 1) and co-stimulation (signal 2). However, pre-exposure of CD8 T cells to IFN-inducers such as viruses or poly(I∶C) prior to antigen signaling is inhibitory, indicating that the timing of IFN exposure is of essence. We show here that CD8 T cells pretreated with poly(I∶C) down-regulated the IFN receptor, up-regulated suppressor of cytokine signaling 1 (SOCS1), and were refractory to IFNβ-induced signal transducers and activators of transcription (STAT) phosphorylation. When exposed to a viral infection, these CD8 T cells behaved more like 2-signal than 3-signal T cells, showing defects in short lived effector cell differentiation, reduced effector function, delayed cell division, and reduced levels of survival proteins. This suggests that IFN-pretreated CD8 T cells are unable to receive the positive effects that type 1 IFN provides as a signal 3 cytokine when delivered later in the signaling process. This desensitization mechanism may partially explain why vaccines function poorly in virus-infected individuals.

## Author Summary

Vaccines are used to protect individuals against infection with a number of different pathogens and depend on the formation of antigen specific memory cells. The efficacy of vaccines can be affected by a number of different factors. It has been known for some time now that suppression of the immune system occurs during acute viral infections. Thus, receiving a vaccine during an acute illness may reduce the efficacy of the vaccine administered. We have identified a common mechanism of immune suppression that may occur with many different pathogens that induce a particular inflammatory response. Any pathogen that induces type 1 interferon could potentially suppress the immune response to a subsequent pathological insult. The mechanism of immune suppression identified here was not having a direct negative effect on lymphocytes, but rather was inhibiting the cells ability to receive positive signals that influence their differentiation, expansion and memory formation. This desensitization mechanism may partially explain why vaccines function poorly in virus-infected individuals.

## Introduction

The fact that virus infections can induce a transient state of immune suppression was first described over a century ago, as patients acutely infected with the measles virus failed to develop a recall response to tuberculin even though they had previously been immunized [1]. Since then, infection with a number of other viruses, including HIV [2], Lymphocytic choriomeningitis virus (LCMV) [3], CMV [4] and Influenza A [5] have been shown to induce a transient state of immune suppression in humans and animal models [6], [7]. Although virus-induced immune suppression can affect many aspects of the immune system, it is often associated with a reduced ability of T cells to proliferate in response to mitogens or antigen-specific stimulation. Viruses may induce this suppression by directly infecting cells of the immune system, but they can also induce immune suppression without directly targeting hematopoietic cells. *In vitro* studies have shown that inhibition of T cell proliferation can be due to death receptor-associated activation-induced cell death (AICD) [8], [9], impaired antigen presentation [10], [11], exposure to immunosuppressive cytokines [12], and perhaps to competition for limited amounts of cytokine growth factors. Recent *in vivo* studies from our laboratory showed that type 1 IFN can be a profound and universal factor inducing suppression of T cell proliferation during viral infections if the T cells are exposed to type 1 IFN prior to encountering their cognate ligand [13].

Efficient clonal expansion and differentiation of CD8 T cells is required to develop protective memory CD8 T cells. This requires three signals: a cognate peptide MHC-TCR interaction (signal 1), co-stimulation (signal 2), and infection-induced cytokines (signal 3) [14]–[16]. CD8 T cells that encounter antigen and co-simulation undergo programmed cell division, but these two signals alone are not sufficient for full effector cell differentiation and survival into memory [14], [17], [18]. CD8 T cells need a third signal, provided by cytokines, including IL-12 or type 1 IFN, for efficient clonal expansion, differentiation into various effector populations, acquisition of cytolytic effector functions, and memory formation [15], [19]. One *in vitro* study showed that without IL-12, CD8 T cells did not proliferate well or develop full effector function [20]. Type 1 IFN, however, can evidently substitute for IL-12 as a signal 3 cytokine [21], [22].

Signal 3 cytokines are required for efficient clonal expansion in response to antigen, and the infecting pathogen and resulting inflammatory environment determine which cytokine(s) provide signal 3 activity [23]–[26]. LCMV-specific CD8 T cells use type 1 IFN as the signal 3 cytokine for effective primary T cell expansion [25], [27], [28], whereas Listeria and VSV depend on both type 1 IFN and IL-12 [23], [25], [29], [30]. Studies showed that IFNαβ Receptor (R) KO LCMV-specific transgenic P14 CD8 T cells divided similarly to WT P14 cells but had reduced survival, thereby limiting their overall clonal expansion [27]. In other systems, the addition of adjuvants or IL-12 to activated CD8 T cells promoted their expansion by up-regulating the IκB family member BCL3, which was found to prolong T cell survival [31]–[33].

Signal 3 cytokines also play an important role in CD8 T cell differentiation into various phenotypic and functional effector populations. Differences in CD8 T cell exposure to co-stimulatory molecules and cytokines can alter their differentiation into early effector cells (EECs), short-lived effector cells (SLECs) and memory precursor effector cells (MPECs) [17], [25], [34]. Recent studies investigating the role for signal 3 cytokines in primary CD8 T cell responses have shown that loss of IL-12R, IFNAR, or both receptors can alter CD8 T cell differentiation, showing reduced SLEC and increased MPEC formation [25], [35]. Signal 3 cytokines have also been shown to augment the acquisition of CD8 T cell effector functions, including production of cytokines (IFNγ and TNF) and CTL activity. *In vitro* studies showed that without type 1 IFN or IL-12, CD8 T cells had decreased lytic ability and low levels of granzyme B expression [21], [27], [36]. Additional *in vivo* studies showed reduced granzyme B expression in IFNαβR KO transgenic P14 CD8 T cells compared to WT P14 CD8 T cells [27]. However, not all infection models show the same requirements for the specific signal 3 cytokines in driving these effector functions [25], [27].

Type 1 IFN signaling is complex in that it can activate multiple downstream pathways, including the JAK/STAT pathway. Engagement of the type 1 IFN receptor promotes phosphorylation of downstream STAT molecules, including STAT1, 3, 4, and 5 [37]. The combination of STAT molecule(s) that are phosphorylated and translocated into the nucleus controls the outcome of CD8 T cell activation. Activation of STAT1 downstream of type 1 IFN receptor signaling generally has anti-proliferative effects on CD8 T cells [38], [39]. In contrast, type 1 IFN-mediated activation of STAT3 and/or STAT5 has anti-apoptotic and pro-mitogenic effects [38], [40], [41]. Type 1 IFN signaling via STAT4 promotes both the acquisition of effector function, including IFNγ production, and clonal expansion [21], [42]. Recent studies showed that during LCMV infection, virus-specific CD8 T cells had decreased total STAT1 levels and increased STAT4 levels, thereby promoting effector T cell differentiation and clonal expansion over anti-proliferative effects [38], [43]. Thus, type 1 IFN can have both inhibitory and stimulatory effects on CD8 T cell proliferation, and when type 1 IFN provides signal 3 cytokine activity, it has positive effects on CD8 T cell expansion.

The timing of exposure of CD8 T cells to all 3 signals is very important, as T cells exposed to virus-induced inflammatory environments prior to cognate antigen respond differently to signals 1 and 2 compared to CD8 T cells from naïve environments [13], [44], [45]. Under circumstances when CD8 T cells see antigen and co-stimulation prior to or at the same time as inflammatory cytokines, IL-12 or type 1 IFN have positive effects on T cell differentiation and expansion. However, CD8 T cells pre-exposed to virus-induced inflammatory environments showed reduced proliferation when exposed to cognate antigen [13], [44]. Virus-induced impaired proliferation could be mimicked by the type 1 IFN-inducer poly(I∶C). In this study, we utilized poly(I∶C) to study the mechanism of IFN-mediated virus-induced T cell immune suppression. We sought to investigate whether the IFN-mediated suppression of CD8 T cells is due to type 1 IFN having direct suppressive effects on CD8 T cells or if it inhibits the positive effects IFN has on CD8 T cells. We show here that poly(I∶C)-pretreated CD8 T cells are refractory to IFNβ signaling in terms of downstream STAT phosphorylation, suggesting that they are unable to receive positive effects that signal 3 cytokines normally provide during acute infections. Indeed, these out-of-sequence signal 3 CD8 T cells were found to behave more similar to 2-signal-only CD8 T cells rather than T cells that receive all 3 signals in the proper order. Therefore, the inability to respond to signal 3 cytokines limits CD8 T cell expansion and suggests a causative mechanism for reduced vaccine efficacy when administered during acute infections.

## Results

### Poly(I∶C)-induced sensitization to impaired proliferation is transient and requires direct effects of type 1 IFN

**Figure 1:**
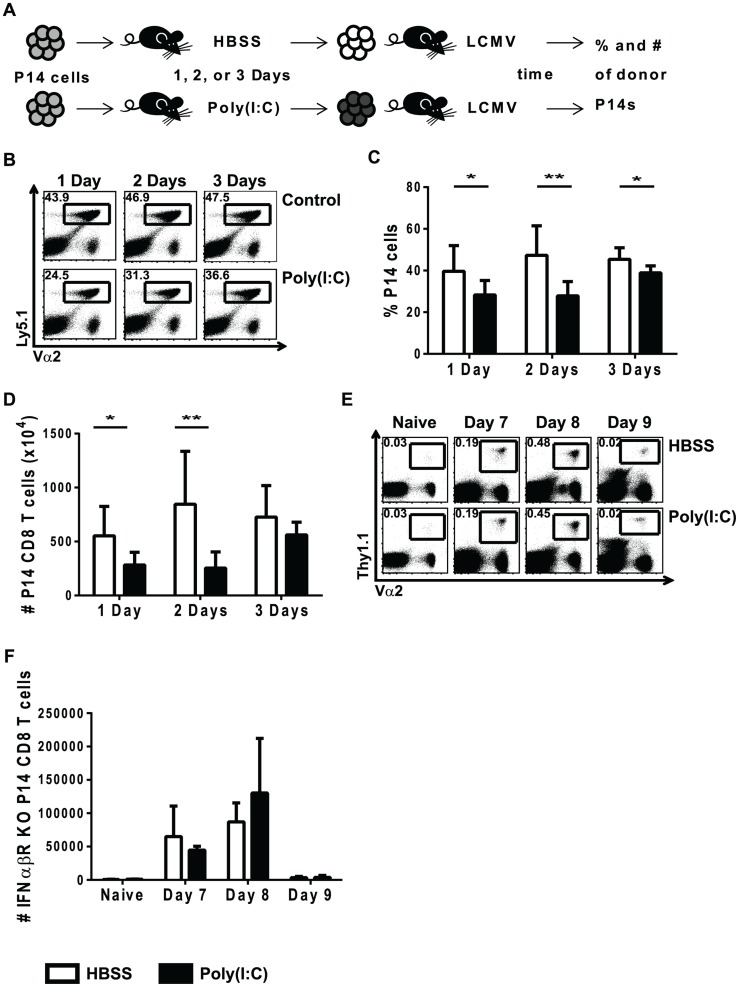
Poly(I∶C)-induced impaired proliferation is transient and requires direct effects of type 1 IFN. (A) Experimental design for poly(I∶C)-induced suppression of proliferation. P14 transgenic CD8 T cells were adoptively transferred into Thy1.2+/Ly5.2+ congenic mice. Recipient mice were inoculated with HBSS or poly(I∶C) for different amounts of time (1, 2, or 3 days), and then splenocytes were isolated and the total frequency of donor P14 cells was determined by flow cytometry in order to transfer equal numbers of transgenic cells into separate congenic recipients. These host mice were then infected with 5×10^4^ pfu of LCMV and harvested at different days post-infection to determine frequency, number, and function. (B) P14 transgenic T cells were identified based on Ly5.1+Vα2+CD8α+ cells. Representative flow cytometry plots of cells harvested at day 7 post LCMV infection, gated on CD8α+ cells show frequency of P14 cells that have been HBSS- or poly(I∶C)-pretreated for 1, 2, or 3 days. Frequency (C) and total number (D) of poly(I∶C)(black bars)- or HBSS(open bars)-treated cells for 1, 2 or 3 days of pretreatment harvested at day 7 post LCMV infection is graphed. (E–F) IFNαβR KO P14 transgenic CD8 T cells transferred into WT congenic mice before poly(I∶C) or HBSS treatment. Equal numbers of IFNαβR KO P14 cells were transferred into separate mice subsequently inoculated with LCMV and harvested at day 7, 8, or 9 post infection. (E) IFNαβR KO P14 cells were identified by Vα2+Thy1.1+ cells in the representative flow cytometry plots gated on CD8α+ cells. (F) Total number of IFNαβR KO P14 cells calculated at different time points post LCMV infection. Data combined from 2 independent experiments, with n of 4 mice per group (C and D), and are representative of 3–4 experiments harvested at different days post LCMV infection with n of 3–4 mice per group (E and F).

Previously, we had shown that CD8 T cells exposed to exogenous cognate antigen 3–9 days, but not 12 days, after initiation of a virus infection proliferated poorly in response to a cognate antigen stimulus, and viruses that induced a strong type 1 IFN response had the greatest suppressive effects [13]. To investigate the mechanism of this virus-induced suppression of T cell proliferation, the IFN-inducer poly(I∶C) was used to prime CD8 T cells. Congenic transgenic LCMV-specific P14 CD8 T cells were used here to study virus-specific T cells exposed to the IFN-inducer poly(I∶C) prior to infection. As demonstrated in **Figure 1A**, Ly5.1 P14 cells were transferred into Ly5.2 B6 hosts that were inoculated with either HBSS or poly(I∶C). One to three days later, splenocytes were isolated, and equal numbers of P14 cells (enumerated by flow cytometry staining) were transferred into recipients that were immediately infected with LCMV. Spleens from recipient mice were harvested at the peak of transgenic T cell expansion, day 7 post infection, and the proportion (**Figure 1B and 1C**) and total number (**Figure 1D**) of transgenic P14 cells were then determined. Suppression of proliferation of poly(I∶C)-pretreated P14 cells (black bars) was greatest at 1 and 2 days of treatment compared to control treated cells (open bars). After 3 days of pre-treatment, clonal expansion of poly(I∶C)-pretreated P14 cells was comparable to that of the HBSS-pretreated control P14 cells, indicating that poly(I∶C)-mediated suppression of proliferation is a transient effect.

Because type 1 IFN is required for efficient clonal expansion of LCMV-specific CD8 T cells, we investigated its role in the reduced proliferation seen in poly(I∶C)-pretreated CD8 T cells. We previously showed that impaired proliferation required direct effects of type 1 IFN acting on the T cell [13], and this is illustrated in **Figure 1E**, using a similar experimental set up as that described in **Figure 1A**. Congenic Thy1.1 IFNαβR KO P14 CD8 T cells were transferred into Thy1.2 mice that were inoculated with either HBSS or poly(I∶C). One day later, equal numbers of transgenic T cells were transferred into mice prior to LCMV infection. The percentage (**Figure 1E**) and total number (**Figure 1F**) of donor IFNαβR KO P14 cells in recipient host mice were determined at various times after LCMV infection. Poly(I∶C)-pretreated IFNαβR KO P14 cells proliferated to similar numbers as the control-treated counterparts at both day 7 and 8 post LCMV infection. The fact that these CD8 T cells lacked expression of the IFNαβR and showed no difference in proliferation between HBSS- and poly(I∶C)-primed groups suggested that there was a direct role for type 1 IFN on the CD8 T cells in this model of immune suppression.

### Poly(I∶C)-pretreated CD8 T cells are transiently refractory to IFNβ stimulation in terms of STAT phosphorylation

**Figure 2:**
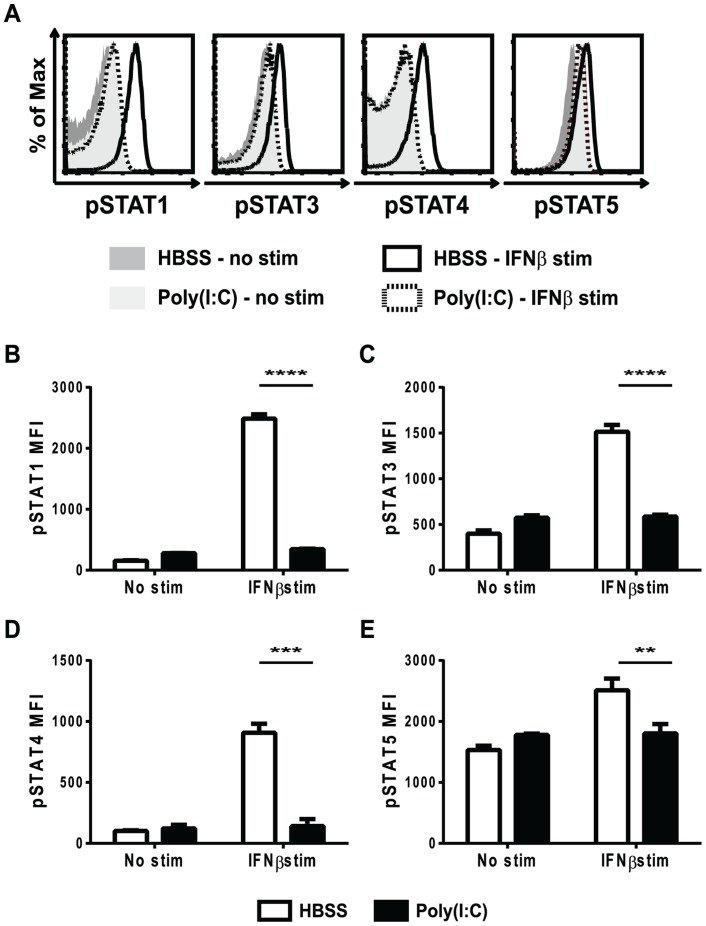
Poly(I∶C)-pretreated CD8 T cells are refractory to IFNβ stimulation in terms of STAT phosphorylation. Mice were HBSS- or poly(I∶C)-treated for 1 day. Splenocytes were isolated and either unstimulated, or stimulated *ex vivo* with IFNβ for 30 min and then stained for (B) pSTAT1,(C) pSTAT3,(D) pSTAT4, and (E) pSTAT5. (A) shows representative histograms gated on CD44lo CD8α+ (naïve) T cells showing pSTAT1, 3, 4 or 5 staining in HBSS– or poly(I∶C)-pretreated naïve CD8 T cells unstimulated (shaded histograms) or IFNβ stimulated (open histograms). HBSS-pretreated naïve CD8 T cells stimulated with IFNβ shown as solid line histograms, and poly(I∶C)-pretreated naïve CD8 T cells stimulated with IFNβ shown as dotted line histogram. (B–E) show pSTAT MFI of naive T cells from unstimulated vs. IFNβ stimulated cells HBSS (open bars) or poly(I∶C) (black bars) pretreated for 1 day. Data are representative of at least 4 independent experiments with n of 3 mice per group.

Knowing that type 1 IFN delivered at an optimal time can provide a positive signal 3 to CD8 T cells and enhance their proliferation, we questioned whether an out of sequence early exposure to IFN would interfere with later attempts at IFN signaling. Type 1 IFN can activate multiple downstream STAT molecules including STAT1, 3, 4, and 5. Because type 1 IFN can have both positive and negative effects on T cell expansion, where recent studies have shown that the specific STAT(s) activated dictate the outcome, all of the aforementioned STAT molecules were tested. The phosphorylation of STAT molecules downstream of the type 1 IFN receptor was thus examined in CD8 T cells from mice pretreated with either HBSS or poly(I∶C). Mice were inoculated with either HBSS or poly(I∶C) for 1 day, and their splenocytes were isolated and stimulated *ex vivo* with mouse IFNβ for ∼30 min, followed by phosflow to examine downstream STAT phosphorylation (**Figure 2**). In unstimulated (non-IFNβ-treated) CD8 T cells, there was very little phosphoSTAT staining, regardless of the pretreatment regimen (**Figure 2A**, shaded histograms). In T cells from HBSS-treated mice (open bars), the phenotypically naïve CD44lo CD8 T cells responded strongly to IFNβ stimulation and showed phosphoSTAT 1, 3, 4 and 5 staining well above the unstimulated controls (solid line, open histograms in **Figure 2A**; open bars in **Figure 2B–E**). However, CD44lo CD8 T cells from mice pre-exposed to poly(I∶C) for 1 day were unable to respond to IFNβ stimulation and did not phosphorylate any downstream STAT molecules tested (dashed line open histogram in **Figure 2A**; black bars in **Figures 2B–2E**). Similar to naïve CD8 T cells, which represent most of the T cells and which are the focus of this study, CD44hi memory phenotype CD8 T cells from poly(I∶C)-pretreated mice also showed reduced response to IFNβ stimulation in terms of downstream STAT phosphorylation (**Figure S1**). Since STAT phosphorylation is a transient event, a kinetic analysis of STAT phosphorylation in cells from HBSS- or poly(I∶C)-inoculated mice stimulated with IFNβ for times ranging from 5 minutes to 2 hours was performed. The poly(I∶C)-pretreated CD8 T cells did not phosphorylate downstream STATs above unstimulated controls at any time point tested (data not shown). The lack of IFNβ- induced phosphoSTAT staining in poly(I∶C)-pretreated T cells suggests that the T cells are unable to respond to IFN and therefore do not receive either the positive or the negative effects that type 1 IFN can have on lymphocytes.

**Figure 3:**
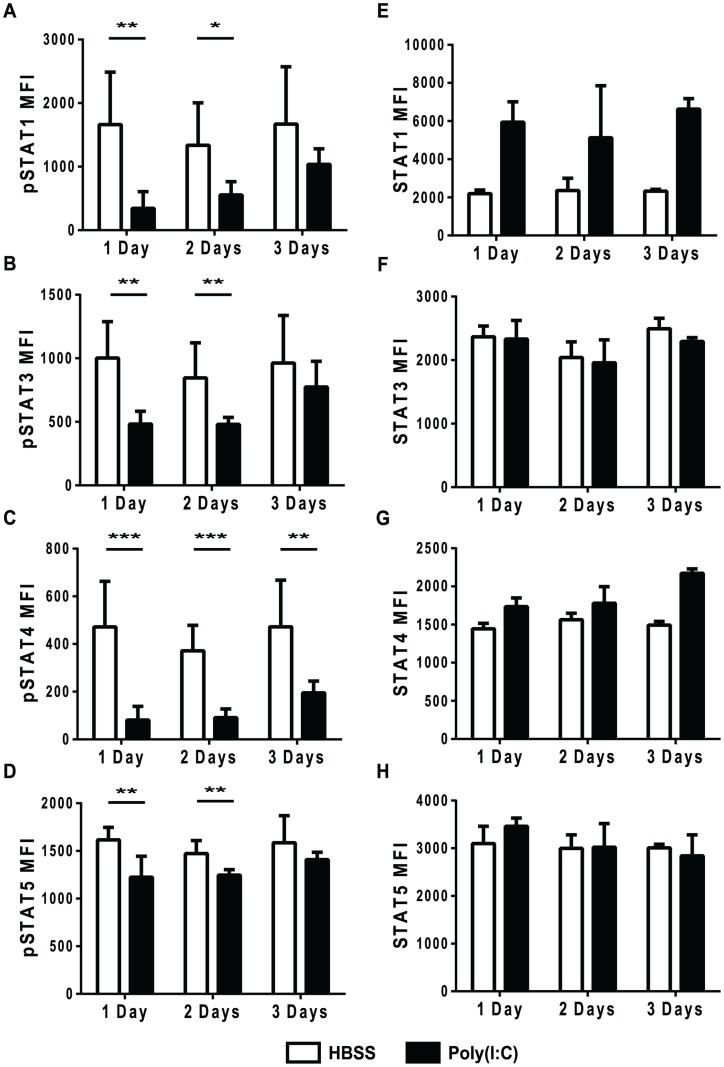
Refractoriness to IFNβ stimulation is transient and not due to reduced total STAT levels. (A–H) Mice were HBSS- (open bars) or poly(I∶C)- (black bars) treated once 1, 2, or 3 days prior to isolation. (A–D) Splenocytes were isolated, stimulated *ex vivo* with IFNβ for 30 min and then stained for phosphoSTATs. Cells were gated on CD44loCD8α+ T cells plotting pSTAT MFI after IFNβ stimulation showing MFI for (A) pSTAT1, (B) pSTAT3 (C) pSTAT4 or (D) pSTAT5. (E–H) Splenocytes were stained *ex vivo* for total STAT proteins including (E) STAT1, (F) STAT3, (G) STAT4, and (H) STAT5. Plots showing total STAT MFI, gated on CD44loCD8α+T cells. Data are representative of 2 independent experiments with n of 3 mice per group.

To test the duration of this unresponsiveness to IFNβ stimulation, mice were inoculated with HBSS or poly(I∶C), and after 1, 2, or 3 days, their splenocytes were stimulated *ex-vivo* with IFNβ for ∼30 min before staining for phosphoSTATs. As shown in **Figure 2**, the phenotypically naive CD8 T cells from mice pretreated with poly(I∶C) for 1 day did not respond to IFNβ stimulation in terms of STAT phosphorylation and this is also shown in **Figures 3A–D** (black bars). Similarly, CD8 T cells from poly(I∶C)-pretreated mice were also less responsive to IFN stimulation when treated 2 days previously compared to controls. However, by 3 days after pretreatment, the CD44lo CD8 T cells from poly(I∶C)-treated mice started to regain the ability to respond to IFNβ stimulation and showed downstream STAT phosphorylation above unstimulated controls. At 3 days, the poly(I∶C)-pretreated CD8 T cells phosphorylated downstream STATs to similar levels as HBSS-pretreated CD8 T cells for all STATs tested except STAT4. Memory phenotype CD44hi CD8 T cells pretreated with poly(I∶C) showed a similar transient unresponsiveness to IFNβ stimulation as the naïve CD44lo CD8 T cell response seen in Figure 3 (data not shown). These data show that the refractoriness to IFNβ stimulation is transient, with kinetics similar to that of the poly(I∶C)-induced impaired proliferation (**Figure 1**).

To make sure that STAT molecules were available to be phosphorylated, total STAT protein levels in naïve CD8 T cells after different days post HBSS or poly(I∶C) inoculation were determined (**Figure 3E–H**). After 1, 2, and 3 days of treatment, total STAT 1, 3, 4 and 5 levels in poly(I∶C)-pretreated naïve CD8 T cells were similar to, if not higher than, the control-treated cells. Total STAT1 expression was higher in poly(I∶C)-pretreated naïve CD8 T cells after 1 day and stayed high through day 3 of treatment, as compared to STAT1 levels in HBSS-treated CD8 T cells. Since STAT1 is an IFN-inducible gene [46], higher STAT1 protein expression in poly(I∶C)-pretreated CD8 T cells was expected. These data indicate that the reduced phosphoSTAT staining found in IFNβ-stimulated poly(I∶C)-pretreated CD8 T cells was not due to lower levels of total STAT protein.

### Poly(I∶C)-pretreated CD8 T cells are responsive to cytokines other than type 1 IFN

**Figure 4:**
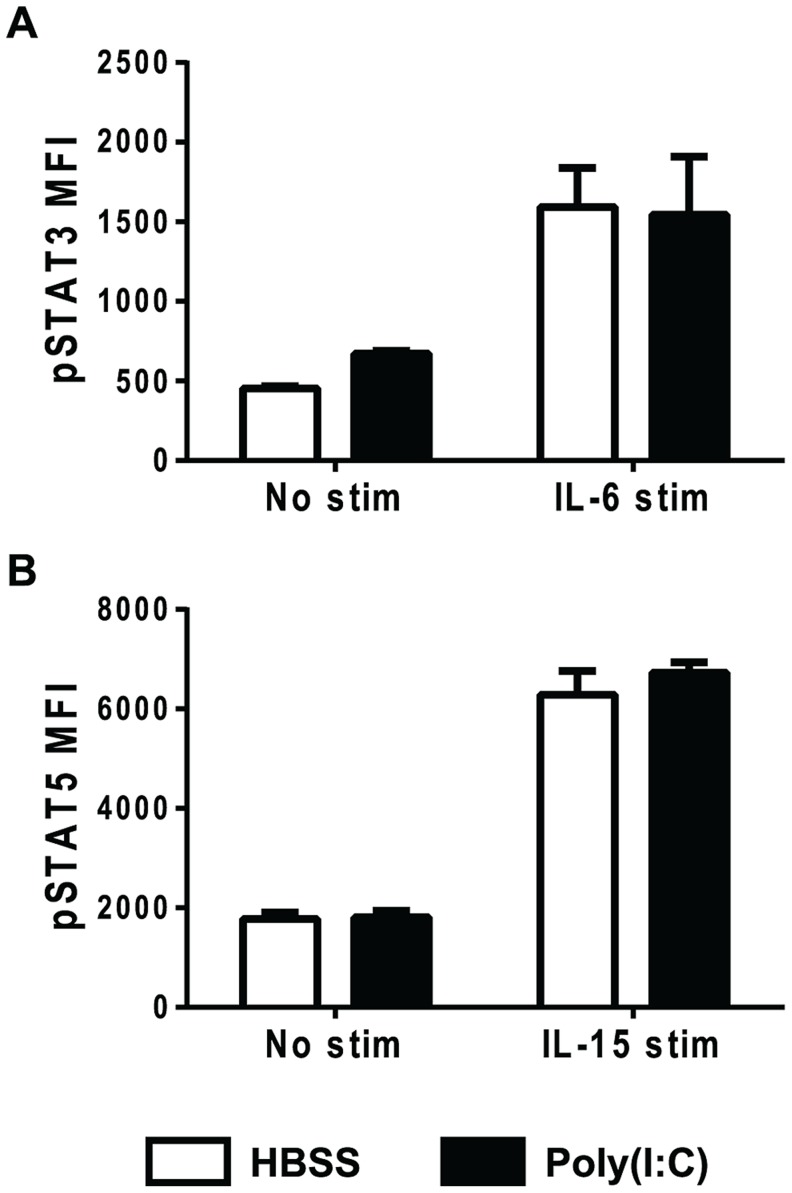
Poly(I∶C)-pretreated CD8 T cells can respond to other cytokines, including IL-6 and IL-15. Mice were inoculated with HBSS (open bars) or poly(I∶C) (black bars) for 1 day. Splenocytes were isolated and either unstimulated, stimulated with IL-6 (A) or IL-15 (B) and then stained for downstream pSTAT3 (A) or pSTAT5 (B). Splenocytes were gated on CD44lo CD8α+ T cells, and plotted for pSTAT MFI. Data are representative of at least 2 independent experiments with n of 3 mice per group.

To test if the poly(I∶C)-primed CD8 T cells were unresponsive to other cytokines, splenocytes were stimulated *ex vivo* with various cytokines for ∼30 min before staining for the appropriate downstream phosphoSTATs. We tested IL-2, IL-6, IL-7, IL-12, and IL-15, because these cytokines have positive effects on T cell survival or proliferation. IL-2, IL-7, and IL-12 stimulation did not elicit positive phosphoSTAT staining in the control- or poly(I∶C)-treated naïve CD8 T cells (**Figure S2**). Of these cytokines tested, IL-6 and IL-15 were found to elicit positive phosphoSTAT staining in HBSS-treated naïve CD8 T cells (open bars) above the unstimulated control levels (**Figure 4A and B**). However, unlike poly(I∶C)-pretreated naïve CD8 T cells stimulated with IFNβ, poly(I∶C)-pretreated naïve CD8 T cells stimulated with IL-6 (black bars, **Figure 4A**) or IL-15 (black bars, **Figure 4B**) responded just as well, in terms of phosphorylating downstream STAT3 and STAT5, respectively, as their control-treated counterparts. Similar to naïve T cells, CD44hi CD8 T cells also phosphorylated downstream STAT3 and STAT5 in response to IL-6 and IL-15 stimulation (respectively) from both the HBSS- and poly(I∶C)-treated groups (**Figure S3**). Together, these data indicate that poly(I∶C) treatment did not make P14 CD8 T cells universally unresponsive to all cytokines; rather, the impairment was instead more specific to type 1 IFN. These data suggest that poly(I∶C)-pretreated CD8 T cells, when put into hosts subsequently infected with LCMV, are not able to respond to the type 1 IFN induced by the virus, and thus are unable to receive positive signal 3 cytokine signals.

### Poly(I∶C)-pretreated CD8 T cells have decreased IFNAR1 and increased SOCS1 expression

**Figure 5:**
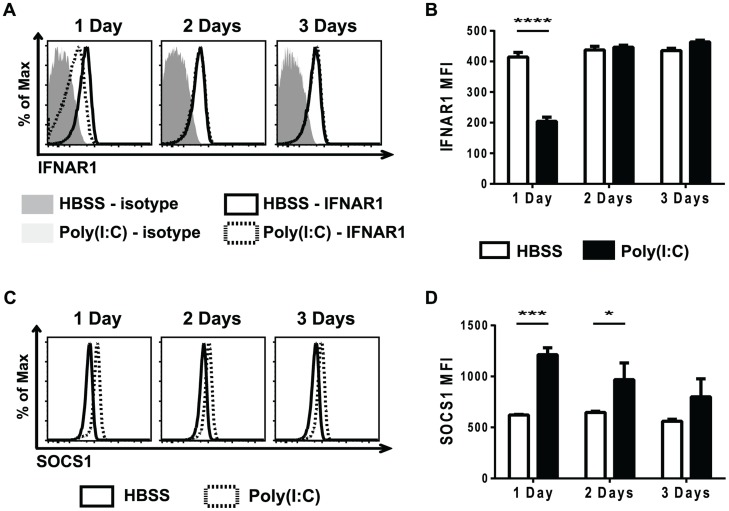
Poly(I∶C)-pretreated CD8 T cells have decreased type 1 IFN receptor expression and increased SOCS1 expression. Mice were HBSS- (open bars) or poly(I∶C)- (black bars) treated for 1, 2, or 3 days. Splenocytes were isolated and stained for type 1 IFN receptor expression (A–B) or SOCS1 expression (C–D). (A) Representative histograms showing IFNAR1, gating on CD44lo CD8α+ T cells and IFNAR1 MFI (B). (C) Representative histograms of SOCS1 expression, gating on CD44lo CD8α+ T cells. (D) SOCS1 MFI in poly(I∶C)- or HBSS-treated cells after 1, 2, or 3 days of pretreatment. Data are representative of 2 independent experiments with n of 3 mice per group.

Cytokine signaling must be tightly regulated in order to prevent over-active and prolonged immune activation. A number of different mechanisms are in place to limit cytokine signaling, including reducing cytokine receptor expression, down-regulating expression of signaling protein components, and up-regulating the expression of suppressors of cytokine signaling proteins (SOCS) 47,48. Reduced STAT protein levels did not account for the refractoriness to IFNβ simulation seen in the poly(I∶C)-pretreated CD8 T cells (**Figure 3**). To investigate why naïve CD8 T cells pre-exposed to poly(I∶C) were unresponsive to type 1 IFN, but not all cytokines, cytokine receptor expression was determined. At various days post HBSS or poly(I∶C) treatment, naïve CD8 T cells were assessed for cytokine receptor signaling components, including portions of the IL-2, IL-6, IL-7, and IL-15 complexes, and these are represented in **Figure S4**. The type 1 IFN receptor is comprised of two components, IFNAR1 and IFNAR2 [37]. Naïve CD8 T cells from mice inoculated with poly(I∶C) for one day had much lower expression levels of IFNAR1, compared to the HBSS-treated cells (**Figure 5A–5B**). However, IFNAR1 expression levels returned to control-treated levels by 2 days post treatment. The CD44hi CD8 T cells had similar kinetics of IFNAR1 expression as the naïve CD8 T cells, showing slightly reduced receptor expression with 1 day treatment of poly(I∶C) but not 2 or 3 days of treatment (**Figure S5**). Thus, unresponsiveness to type 1 IFN at day 1 correlated with the lack of expression of the IFN receptor.

Since poly(I∶C)-pretreated CD8 T cells were still less responsive to IFNβ stimulation, as measured by STAT phosphorylation, 2 days after poly(I∶C) treatment (**Figure 3**), there were likely other suppressive mechanisms to limit IFNβ responsiveness, in addition to the reduced receptor expression shown in **Figure 5A and B**. SOCS proteins are known to inhibit cytokine receptor signaling by acting at many different steps in the JAK/STAT signaling pathway [47]. SOCS1 inhibits type 1 IFN signaling by binding to the receptor-associated JAK protein TYK2, thus blunting IFN receptor signaling [49]. Naïve CD44lo CD8 T cells that were sorted from mice inoculated with HBSS or poly(I∶C) for 1 day showed a 7-fold relative increase in *SOCS1* message expression after poly(I∶C) treatment. We next utilized the well-established protocol for staining for phosphorylated proteins to identify intracellular levels of SOCS1. SOCS1 expression was determined in naïve CD8 T cells from poly(I∶C)- or HBSS-inoculated mice 1, 2, or 3 days after treatment (**Figure 5C–D**). Indeed, at both 1 and 2 days after poly(I∶C) treatment, naïve CD8 T cells had higher expression of SOCS1 compared to the control-treated cells. However, by 3 days of pretreatment, there was no longer a significant difference in SOCS1 expression between control and poly(I∶C)-treated CD8 T cells. CD44hi CD8 T cells also showed increased SOCS1 protein levels (**Figure S5**). This suggests that a combination of a decrease in IFNαβR expression and an increase in SOCS1 expression may account for the observed refractoriness to IFNβ stimulation. These results correlated kinetically with refractoriness to IFNβ stimulation (**Figure 3**) and to the suppressed proliferation seen in poly(I∶C)-pretreated CD8 T cells (**Figure 1**). These experiments do not definitively parse out the relative contributions of decreased receptor expression vs. inhibitory molecule contribution to T cell refractoriness to IFN stimulation, but the results are very consistent with previous work in more tractable systems studying the mechanism of unresponsiveness to IFN [49]–[51]. Further, they clearly show that this unresponsiveness is not due to decreases in overall STAT protein expression.

### Poly(I∶C)-pretreated CD8 T cells behave similarly to 2-signal-stimulated rather than 3-signal-stimulated CD8 T cells

Because the suppression of proliferation of poly(I∶C)-pretreated CD8 T cells correlated with refractoriness to IFNβ stimulation, we hypothesized that poly(I∶C)-pretreated CD8 T cells were unable to receive the positive effects that type 1 IFN exerts as a signal 3 cytokine when delivered in the proper sequence. This hypothesis would suggest that poly(I∶C)-pretreated P14 cells would behave similarly to 2-signal only CD8 T cells, rather than 3-signal CD8 T cells. Thus, we examined their effector phenotype and their abilities to divide, produce cytokines, degranulate, and express the survival protein BCL3 in response to antigen exposure.

**Figure 6:**
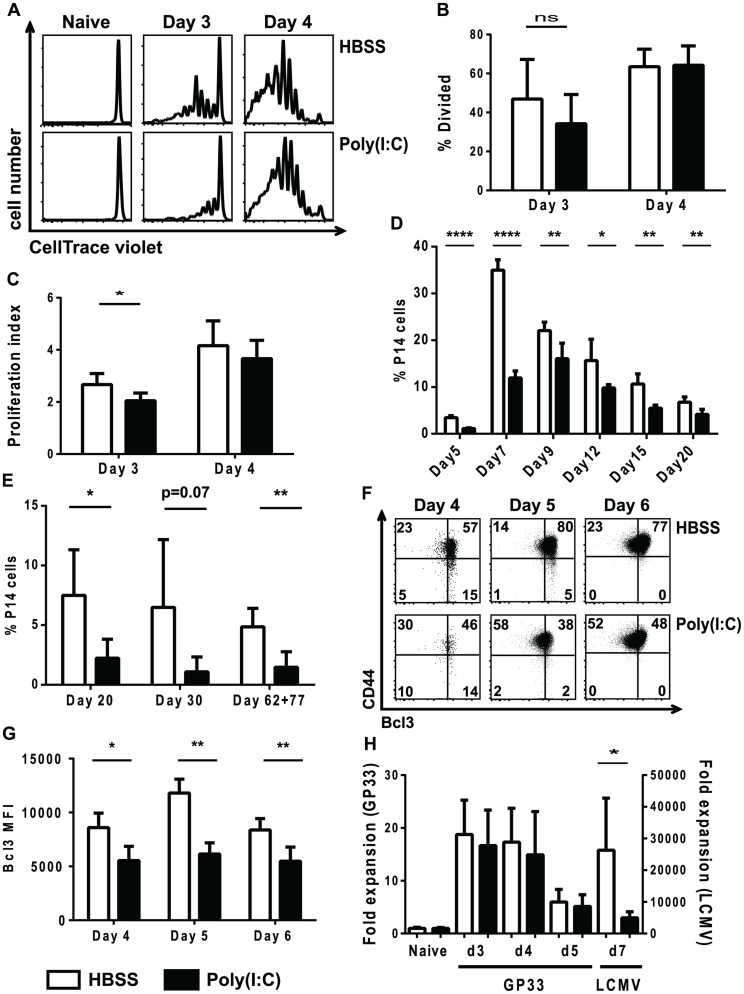
Poly(I∶C)-pretreated P14 CD8 T cells have a delay in division and reduced BCL3 expression. (A–C) Congenic transgenic P14 mice were HBSS or poly(I∶C) treated. One day after treatment P14 cells were labeled with CellTrace Violet, and similar numbers of HBSS- and poly(I∶C)-pretreated cells were transferred into the same recipients, which were subsequently inoculated with LCMV. (A) Representative CellTrace Violet dilution profiles shown for different days post infection. Percent divided (B) and proliferation index (C) of P14 cells pretreated with HBSS (open bars) or poly(I∶C) (black bars) at day 3 and 4 post infection. (D–G) Equal numbers of HBSS (open bars) - and poly(I∶C) (black bars)-pretreated P14 CD8 T cells transferred into the same or different recipients which were subsequently infected with LCMV. (D–E) Spleens were harvested at days 5, 7, 9, 12, 15, 20, 30, 62, or 77 post infection, and the frequency of donor P14 cells of the total CD8 population is graphed. P14 cells were transferred into the same hosts when spleens were harvested from day 5-day 20 post infection and different hosts when harvested at day 30 or later. (F–G) Cells were harvested at day 4, 5, or 6 post LCMV infection. Representative flow cytometry plots showing CD44 and BCL3 expression (F) and BCL3 MFI (G) of the donor P14 cells is graphed. (H) P14 mice were either HBSS (open bars) or poly(I∶C) (black bars) treated for 1 day, after which equal numbers of P14 cells were transferred together into the same recipients that remained uninfected, that received 5 µg 13mer GP_33–45_ peptide, or that were infected with LCMV. Spleens were harvested at days 3–5 post peptide inoculation and/or day 7 post LCMV infection. The appropriate naïve control for was used to calculate the fold expansion. Data are representative of 2 individual experiments with n of 4–6 per group (A–C), one experiment with n of 5 per group (D), 3 individual experiments with n of 4–5 per group (E), and one experiment with n of 3–5 per group (F–G). Data were combined from 3 individual experiments, each with 2 time points tested with n of 5 mice per group (H).

Studies have shown that 2-signal CD8 T cells divide similarly to 3-signal CD8 T cells but have defects in survival [27]. We therefore tested whether the impairment in proliferation seen in poly(I∶C)-pretreated CD8 T cells was due to division or survival defects. A similar experimental setup as shown in **Figure 1A** was used, but after inoculation with HBSS or poly(I∶C), congenic P14 CD8 T cells were labeled with CellTrace violet and transferred together into the same recipients to track cell division during LCMV infection. Cells were harvested at days 3 and 4 post infection, and CellTrace violet dilution was measured. Because we were looking at early days post infection, a larger number of transgenic cells was transferred than what would normally be considered physiologically relevant in order to quantify early cell division. Neither the poly(I∶C)- nor the HBSS-pretreated P14 cells diluted CellTrace violet in naïve mice, indicating that they did not divide (**Figure 6A**). At day 3 post infection, the control-treated P14 cells diluted more CellTrace violet, indicating they had undergone more cell divisions compared to the poly(I∶C)-pretreated P14 cells (**Figure 6A–C**). However, by day 4 post LCMV infection, the division profiles of both HBSS-and poly(I∶C)-pretreated P14 cells appeared similar. Although the percentage of cells that divided was statistically similar between the two groups (**Figure 6B**), the proliferation index of poly(I∶C)-pretreated P14 cells was lower compared to HBSS-treated cells (**Figure 6C**). The proliferation index represents the average number of divisions of the cells that have undergone at least one division. These results show that poly(I∶C)-pretreated CD8 T cells have a delay in the number of cell divisions.

If a delay in cell division were the only thing contributing to the suppression of proliferation, at later time points post infection the expansion of poly(I∶C)-pretreated CD8 T cells might eventually reach the same level as the control-treated cells. Therefore, a time course of HBSS- or poly(I∶C)-pretreated P14 CD8 T cell expansion in response to LCMV infection was performed. The peak of expansion of poly(I∶C)-pretreated P14 CD8 T cells was delayed (day 9) compared to HBSS-pretreated P14 CD8 T cells (day 7), but the magnitude of the response was still reduced in the poly(I∶C)-pretreated cells (**Figure 6D**). In addition, out-of-sequence P14 cells showed decreased memory frequencies (**Figure 6E**) and number (data not shown) compared to their control treated counterparts at multiple time points tested. This suggests that the defects in clonal expansion were not solely due to a delay in cell division but may also be due to other factors such as defects in cell survival. *In vitro* and *in vivo* studies by others found that survival of activated T cells in response to signal 3 cytokines and adjuvants was in part due to an increase in the IκB family member BCL3 and that cells lacking signal 3 cytokines have reduced expression of BCL3 [31]–[33]. To determine if the poly(I∶C)-treated virus-stimulated T cells resembled two signal only T cells in this respect, the expression of BCL3 was thus determined in HBSS-control or poly(I∶C)-pretreated P14 CD8 T cells after LCMV infection. Indeed, a lower percent of poly(I∶C)-pretreated P14 cells up-regulated BCL3 than HBSS-treated P14 cells at days 4, 5 and 6 post LCMV infection (**Figure 6F**). Additionally, the BCL3 MFI of poly(I∶C)-pretreated P14 CD8 T cells was lower than in HBSS control-treated cells (**Figure 6G**). Combined, these results showed that, similar to 2-signal CD8 T cells, out-of-sequence signal 3-stimulated P14 CD8 T cells had a delay in cell division when compared to CD8 T cells that receive all 3 signals in the correct order, and that they had defects in a survival protein that may limit the ability of these cells to clonally expand.

To further support the hypothesis that out-of-sequence signal 3 CD8 T cells do not receive the positive effects that type 1 IFN can have as a signal 3 cytokine during acute virus infection, and thereby contribute to suppression of proliferation, we determined the frequency of poly(I∶C)- or HBSS-pretreated P14 cells after cognate peptide stimulation. Congenic P14 mice were directly treated with HBSS or poly(I∶C) for 1 day, and their splenocytes were transferred together into the same recipients that were naive, that received 13mer GP_33–45_ peptide or that were inoculated with LCMV. Here the LCMV infection should induce high levels of type 1 IFN, whereas the peptides would be poor type 1 IFN inducers. **Figure 6H** shows that at all time points tested, poly(I∶C)-pretreated P14 cells expanded to similar levels as HBSS-pretreated P14 cells in mice that only saw antigen (13mer GP_33–45_) and did not have a major inflammatory response. However, poly(I∶C)-pretreated P14 cells had defects in expansion in response to the IFN-inducing LCMV infection compared to control treated cells (note the different axis for GP33 peptide or LCMV inoculation). Given that poly(I∶C)-pretreated P14 cells expanded to similar levels in response to antigen only but had defects in expansion in response to antigen and inflammation (i.e. live virus infection), these results further support our hypothesis that out-of-sequence CD8 T cells are unable to receive positive effects of signal 3 cytokines during acute infections.

**Figure 7:**
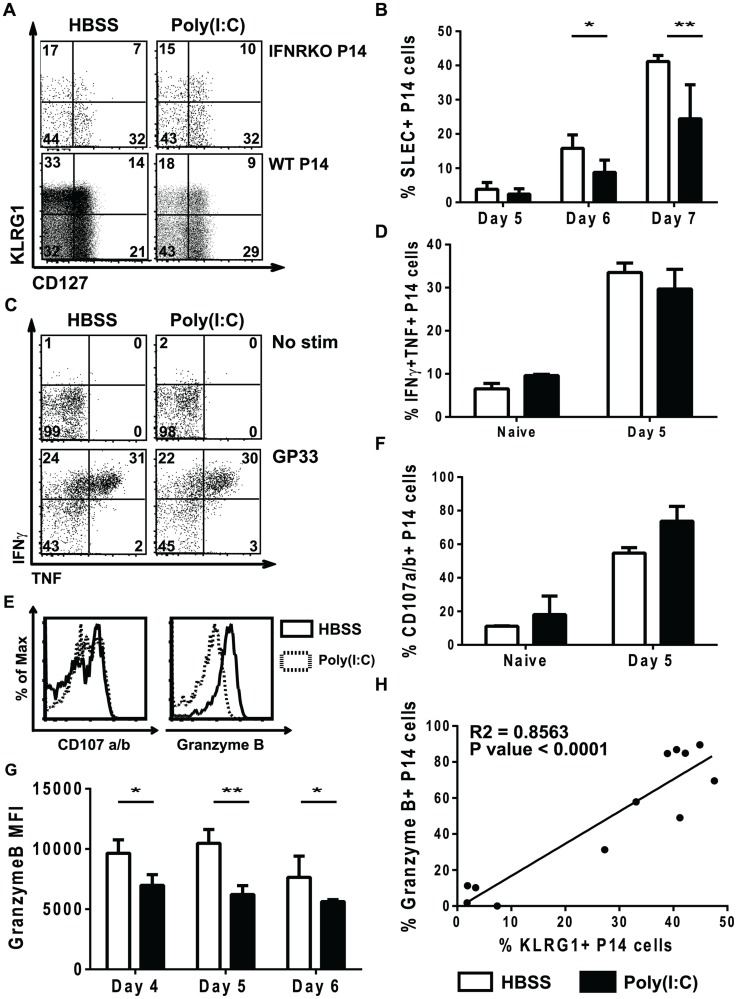
Poly(I∶C)-pretreated P14 CD8 T cells have reduced effector function. (A–B, E–H) WT or IFNαβR KO P14 cells were transferred into congenic mice that were inoculated with HBSS or poly(I∶C), and 1 day later equal numbers of P14 cells were transferred into separate congenic mice subsequently infected with LCMV. (C–D) P14 mice were directly treated with poly(I∶C) or HBSS for 1 day prior to transfer into congenic mice that were infected with LCMV. Cells were harvested at various days post infection (day 4, 5, 6, or 7). (A–C) cells were unstimulated or (C–H) stimulated *ex vivo* with GP33 peptide. (A) Representative flow cytometry plots gating on IFNαβR KO P14 cells (Vα2+Thy1.1+ CD8α+) or WT P14 cells (Vα2+Ly5.1+CD8α+) showing KLRG1 and CD127 expression at day 7 p.i. (gates drawn using naïve CD8 T cells) (B) Percent of WT donor P14 cells that express the SLEC phenotype (KLRG1hi/CD127lo) at different days post infection. (C) Representative flow cytometry plots gating on P14 cells (Vα2+Ly5.1+CD8α+) showing TNF and IFNγ production at day 5 p.i. (D) Frequency of HBSS- (open bars) or poly(I∶C)- (black bars) pretreated P14 CD8 T cells that produce both TNF and IFNγ. (E) Representative histograms for either CD107a/b or granzyme B staining in poly(I∶C)- (open solid lines) or HBSS- (shaded dashed lines) pretreated P14 CD8 T cells. Total frequency of HBSS- (open bars) or poly(I∶C)- (black bars) P14 cells producing CD107a/b (F) or MFI of granzyme B at different time points post infection (G). (H) Correlation between frequency of granzyme B+ P14 cells and KLRG1+ P14 cells at day 5 post infection. Data are representative of at least 2 individual experiments with n of 3–5 mice per group.

Signal 3 cytokines have been shown to regulate the differentiation of CD8 T cells into distinct effector populations including EEC, SLEC and MPEC [17], [24], [34], [35]. Therefore, we examined the ability of poly(I∶C)-pretreated CD8 T cells to differentiate into EEC, SLEC and MPEC populations, which can be distinguished based on expression of KLRG1 and CD127 [30], [34], [52]. A similar experimental model was used as shown in **Figure 1A**, where WT or IFNαβR KO P14 cells were transferred into mice for 1 day of treatment with poly(I∶C) or HBSS prior to a second transfer into congenic hosts that were subsequently inoculated with LCMV. At different days post infection, splenocytes were isolated and stained for KLRG1 and CD127. At day 7 post infection, the IFNαβR KO P14 CD8 T cells had similar proportions of SLEC (KLRG1hi, CD127lo), MPEC (KLRG1lo, CD127hi) and EEC (KLRG1lo, CD127lo), regardless of the pretreatment regime. These data are consistent with other results showing that type 1 IFN is important for SLEC differentiation in various infection models [25], [35]. However, poly(I∶C)-pretreated WT P14 CD8 T cells had reduced proportions of SLEC populations and increased EEC proportions compared to the HBSS-pretreated WT P14 CD8 T cells (**Figure 7A**). This data supports our hypothesis that the out-of-sequence CD8 T cells behave more similar to 2-signal only CD8 T cells (IFNαβR KO P14 cells) in terms of effector cell differentiation. The defect in SLEC differentiation in poly(I∶C)-treated cells can be seen as early as day 5 post infection, but is more dramatic at days 6 and 7 post infection (**Figure 7B**). The proportion of MPECs were generally similar to or slightly elevated in poly(I∶C)-pretreated P14 cells as compared to control-treated counterparts at days 5–7 post infection (data not shown). These data show that in addition to CD8 T cells requiring signal 3 cytokines for proper effector cell differentiation, they also need to see the signals in the appropriate order.

In some infection models, two-signal CD8 T cells can produce similar proportions of cytokines as compared to 3-signal CD8 T cells (VSV), but other infection models show reduced cytokine production (Listeria) [25], [27]. Therefore, we compared poly(I∶C)- and HBSS-treated cells for their ability to produce the effector cytokines TNF and IFNγ. A similar experimental model was used as shown in **Figure 1A**, where P14 cells were transferred into mice for 1 day of treatment with poly(I∶C) or HBSS or P14 mice were treated directly with poly(I∶C) or HBSS prior to transferring cells into congenic hosts that were subsequently inoculated with LCMV. At day 5 post infection, splenocytes were isolated and stimulated *ex vivo* with or without cognate peptide GP33 for 5 hours. Using naïve CD8 T cells, isotype controls and fluorescent minus one staining to distinguish positive vs. negative staining, the poly(I∶C)-pretreated P14 cells produced similar frequencies of TNF and IFNγ compared to control-treated P14 cells after *in vitro* LCMV GP33 peptide stimulation (**Figure 7C**). Plotting the proportion of double cytokine producers (TNF and IFNγ) (**Figure 7D**) revealed no significant difference in the ability of these cells to produce effector cytokines. Using CD107a and b as markers for degranulation, we found that poly(I∶C)-pretreated P14 cells stained to a similar extent, if not slightly more, than HBSS-treated cells in response to GP33 peptide stimulation (**Figure 7E–7F**). However, poly(I∶C)-pretreated P14 CD8 T cells had substantially lower levels of granzyme B expression than control-treated cells at day 5 post infection (**Figure 7E**). Reduced granzyme B expression in the out-of-sequence CD8 T cells was seen as early as day 4 post infection and lasted at least until day 6 post infection (**Figure 7G**). KLRG1 expression in CD8 T cells is considered a marker for effector function, and there was a positive correlation between KLRG1 expression and granzyme B expression (R square = 0.8563, p<0.0001) (**Figure 7H**). Since granzyme B expression has been used as a correlative marker for cytotoxic capability [36], this suggests that poly(I∶C)-pretreated CD8 T cells have reduced cytolytic function compared to HBSS-treated CD8 T cells. These data showing similar cytokine production but reduced granzyme B expression are consistent with published phenotypes for CD8 T cells that only receive 2 signals [25], [27].

A new method to study effector cell function is the trogocytosis assay, whereby target cells are labeled with a membrane dye, mixed with cytotoxic effector cells for 1 hr, and then examined for the transfer of dye to a flow cytometry-defined effector cell population [53], [54]. This is an indicator of how aggressively the effector cells are attacking the targets. **Figure S6** shows that the poly(I∶C)-pretreated P14 cells, 5 days after LCMV infection, had modest but statistically significant reduced ability to acquire the dye from GP33-pulsed RMA cells, when compared to the HBSS-pretreated donor T cells. Reduced incorporation of the lipid dye is an indicator of reduced effector cell function [55], [56].

We tested whether these donor poly(I∶C)-pretreated T cell responses, which were dramatically reduced in number and modestly reduced in effector function, would affect viral load differently than that in mice receiving HBSS-treated cells. Mice receiving either HBSS-pretreated or poly(I∶C)-pretreated P14 CD8 T cells were subsequently infected with LCMV, and viral titers were examined at different time points post infection. As early as day 4 post infection, mice receiving poly(I∶C)-pretreated P14 cells had modest but statistically significant increased viral titers compared to mice receiving control treated P14 cells in the fat pad (4.7±0.1 vs. 4.3±0.08 log pfu, two independent experiments combined for n = 10 per group) respectively (p = 0.0082). In addition, at day 6 post infection, there was a modest but significant increase in viral load in the spleen and liver in mice receiving poly(I∶C)-pretreated P14 CD8 T cells (4.2±0.07 log pfu in the spleen, 4.2±0.08 log pfu in the liver) compared to mice receiving HBSS-pretreated P14 cells (3.8±0.12 log pfu in the spleen, 3.8±0.08 log pfu in the liver) (3 independent experiments combined for n = 14–15, p = 0.0197 (spleen) and p = 0.0074 (liver)). These differences in viral titer are admittedly small, but they are statistically significant and occur in environments where normal endogenous host T cell responses are simultaneously occurring.

## Discussion

Transient states of immune suppression occur during many acute viral infections, and it has long been known that individuals should not get vaccinated when they are sick. Virus-induced immune suppression was first noted over 100 years ago [1], and more recent studies have shown it to be a common element of many viral infections and often be associated with suppressed T cell proliferation in response to antigens and mitogens. *In vitro* studies had suggested that AICD contributed to this inhibition of T cell proliferation [2], [8], [9], [57], and other studies implicated impaired antigen-presenting cell function [10], [11], [58], induction of immunosuppressive cytokines like IL-10, [12], and perhaps the competition for T cell growth factors could play a role. While studying viral infection models, we recently found that a general mechanism of virus-induced immune suppression could be linked to type 1 IFN, normally induced at high quantities in most viral infections [13]. This was somewhat surprising, given that type 1 IFN has been described as a signal 3 cytokine, which drives the expansion and differentiation of T cells after they have encountered cognate ligand (signal 1) and co-stimulation (signal 2). The primary observation of the present report is that if T cells are exposed to type 1 IFN inducers before exposure to cognate ligand, they lose their sensitivity to further IFN stimulation and do not receive the benefits of a signal 3 cytokine. Instead, they behave like T cells receiving only two signals, with defects in effector cell differentiation, reduced effector function, lower expression of a pro-survival protein, and limited clonal expansion.

Dating back to the early days of IFN therapy in humans, it has long been known that lymphocytes like NK cells become hyporesponsive to treatment, and IFN, like many other cytokines, can render treated cells resistant to further IFN stimulation by down regulating the IFN receptor and by inducing factors like SOCS1 that impair IFN-induced signal transduction [47], [48]. We show here that this is the case with virus-specific T cells, and that these T cells pre-exposed to IFN fail to derive the benefit of the positive signal 3 effects of IFN signaling. The implications of this phenomenon are widespread. Because IFN is induced so rapidly during viral infections, one can deduce that the T cells that encounter antigen in the first day or two of infection would respond more impressively than “late-comer” virus-specific T cells stimulated later in infection. Thus, the dynamics of how much antigen is synthesized and presented vs. how much and how quickly IFN is induced may dictate the efficacy of the host response. Secondly, the T cell response to many acute infections, at least in mouse models, is relatively ordered and undergoes a rather synchronized contraction from 6–9 days post-infection. How can this occur when the amount of T cell proliferation is a programmed event [59]–[62] and when different T cells should encounter antigen at different time periods? We would argue that the late-comer T cells, because of their previous exposure to IFN, would not undergo as many divisions and possibly have lower survival properties, thereby enabling them to contract when the rest of the T cells do. Third, we would argue that naïve or memory T cells specific to third party antigens would not respond well to a cognate antigen stimulus if they were first exposed to the IFN milieu of a viral infection and then stimulated with antigen. This failure to respond to recall antigens, such as tetanus toxoid or tuberculin, is a common feature of virus-induced immune suppression in humans, and the weak efficacy of vaccines in already infected individuals may well be a function of the same problem [63]–[65]. Finally, under conditions when a host develops a persistent viral infection there would be a chronic stimulation of the type 1 IFN response, and such hosts would probably not immunologically respond well to either the antigens of the infecting virus or to third party antigens on challenge. This weak response to third party antigens is not only seen during persistent viral infections but also during chronic autoimmune diseases, such as lupus erythematosus, where signal 3 cytokines may be chronically produced [66], [67]. We therefore suggest that the elimination of signal 3 stimulation by out-of-sequence exposure to the signal 3 stimulant, in this case IFN, would be a common factor disrupting T cell responses in the context of acute or persistent viral infections.

This generalized IFN-induced impairment of proliferation is one example of how out-of-sequence signaling can alter responses to cognate antigen exposure. On the other hand, virus-induced inflammatory environments can alter the response of bystander CD8 T cells not specific for the infecting virus to third party cognate antigens by driving the T cells down a different differentiation pathway [45]. Our previous studies showed that transgenic CD8 T cells exposed to virus-induced inflammatory environments were sensitized to undergo rapid effector function such that upon stimulation with cognate antigen they produced cytokines including granzyme B and IFNγ within a few hours and without a need for cell division. The sensitization to rapid effector function was most dramatic with viruses that induced a strong type 1 IFN response, and this event could also be induced by poly(I∶C). We do not know if the CD8 T cells sensitized to rapid effector function are in fact the same cells that ultimately are suppressed in proliferation. However, we do know that these two changes in T cell response to cognate antigen stimulation occur by very distinct mechanisms and occur in virus-induced inflammatory environments; consequently, the impairment of proliferation may contribute to generalized IFN-induced immune suppression, even though there may be an initial transient activation of the T cells.

Type 1 IFN can have both stimulatory and inhibitory effects on CD8 T cell proliferation, but here it was initially unclear if poly(I∶C)-pretreated CD8 T cells were receiving direct inhibitory signals or fewer stimulatory signals from IFN. Since type 1 IFN signaling can act through multiple STATs, each capable of altering cell fate, it might have been expected that poly(I∶C)-pretreated CD8 T cells would have had different STAT phosphorylation in response to IFNβ stimulation. Recent work has shown that virus-specific CD8 T cells down-regulate total STAT1 and up-regulate STAT4, so that when IFN signals though the IFN receptor the anti-proliferative effects of STAT1 will be overcome by the positive effects mediated through STAT4 [38], [43]. Therefore, poly(I∶C)-pretreated CD8 T cells could have had more pSTAT1 and less pSTAT4 than control-treated cells after IFNβ stimulation. However, this was not the case at the time points studied, as phosphorylation of all tested STATs was reduced (**Figures 2** **and** **3**). The fact that all pSTATs were reduced in poly(I∶C)-pretreated CD8 T cells suggested that IFN was not having a direct negative effect other than by desensitizing cells to the positive effects that a later exposure to IFN could mediate.

It should be noted that not only were naïve CD8 T cells unresponsive to IFNβ stimulation after poly(I∶C) treatment, but CD4 T cells and NK cells were also refractory to further IFNβ stimulation in terms of STAT phosphorylation (data not shown). Type 1 IFN has been shown to act directly on CD4 T cells, NK cells and B cells to promote effector function [68]–[70], and these results may indicate that in addition to poly(I∶C) inducing inhibitory effects on CD8 T cell proliferation, it may also have suppressive effects on other lymphocyte populations that utilize IFN at another time for their activation. Indeed, reduced antibody production by B cells and lower NK cell cytotoxicity have been seen under conditions of virus-induced immune suppression [69], [71].

Antigen and co-stimulatory molecules provide proper signals for T cell activation and differentiation, but more recent studies have focused on the role for inflammatory cytokines in these processes. We find here an additional layer of complexity in that the timing of T cell exposure to signal 3 cytokines is extremely important. If CD8 T cells are unable to receive the positive effects of type 1 IFN, as shown in this study, they should behave more like T cells that only received 2 signals, rather than 3 signals. This was the case, as the out-of-sequence signal 3 CD8 T cells had defects in SLEC differentiation and effector function. Poly(I∶C)-pretreated CD8 T cells degranulated, as shown by CD107a/b staining, but they had reduced granzyme B expression (**Figure 7**), suggesting that poly(I∶C)-pretreated CD8 T cells have lower cytolytic capabilities at day 5 post-infection. This is consistent with the phenotype of signal 3-lacking T cells but inconsistent with our observation that prior signaling with IFN can sensitize a CD8 T cell to rapid effector function on exposure to cognate ligand. That enhanced effector function, however, was examined shortly after TCR (a few hours) stimulation and not examined at day 5 post infection. Thus, out of sequence exposure to IFN may initially stimulate effector function of CD8 T cells but not sustain it as they poorly proliferate.

Another hallmark of 2-signal only CD8 T cells is limited clonal expansion, which in many cases is attributed to decreased survival. Although the exact mechanism is unknown, BCL3 prolongs the survival of activated CD8 T cells after signal 3 cytokine addition or CpG adjuvant administration [31]–[33], [72]. The IFN-induced suppression of proliferation seen here may also have been due to a decrease in survival. This idea was supported by poly(I∶C)-pretreated CD8 T cells having lower expression of the pro-survival protein BCL3 (**Figure 6**). We show here, that poly(I∶C)-pretreated P14 cells also had a delay in cell division compared to HBSS-treated cells in response to LCMV infection (**Figure 6**). Interestingly, the delay in cell division of poly(I∶C)-pretreated P14 cells is seen at day 3 post infection, but not at day 4 post infection, matching the kinetics of the timing of the ability of CD8 T cells to respond to IFNβ signals by phosphorylating downstream STATs (**Figure 3**). The positive effects that an inflammatory environment can have on CD8 T cell expansion is also shown here, whereby P14 T cells expanded ∼20 fold in response to GP33 peptide, but expanded more than 30,000 fold in response to LCMV infection. The facts that poly(I∶C)-pretreated P14 cells are suppressed in proliferation in response to LCMV infection but not to GP33 peptide stimulation support the idea that the refractoriness to IFN stimulation contributes to reduced expansion (**Figure 6**). This mechanism of IFN-induced immune suppression may explain how many virus infections can inhibit T cell responses, by limiting the ability of T cells to receive stimulatory effects from the environment.

To summarize, if CD8 T cells see signal 3 first, they become refractory to further IFN stimulation and are unable to receive the positive signals that type 1 IFN can provide when delivered at the proper time after antigen and co-stimulation. This limits their ability to clonally expand, to sustain cytolytic capabilities, and form memory. Our studies show lower proportions and numbers of out-of-sequence CD8 T cells at different stages of memory formation (**Figure 6E**), including as late as 11 weeks post infection. Preliminary data show that the out-of-sequence CD8 T cells that do form memory are able produce similar proportions of cytokines, when stimulated *ex vivo*, compared to memory cells from the control environment (data not shown), but the effectiveness of these memory cells has not been further investigated. The efficacy of an out-of-sequence CD8 T cell memory response to secondary challenge is important to study but is beyond the scope of the paper, whose focus was to examine why IFN causes naïve T cells to function poorly during the context of an acute viral infection. Thus, under circumstances when CD8 T cells can receive positive signals, such as during an infection or vaccination with adjuvants, out-of-sequence signals can have a profound effect on CD8 T cell expansion and activation. This out-of-sequence inhibition of T cell proliferation may account for the more general immune suppression seen in many acute virus infections known to induce type 1 IFN. This mechanism of CD8 T cell suppression would be expected to contribute to the reduced efficacy of vaccines when they are administered during an acute infection.

## Materials and Methods

### Ethics statement

This study was carried out in strict accordance with the recommendations in the Guide for the Care and Use of Laboratory Animals of the National Institutes of Health. The protocol was approved by the University of Massachusetts Medical School Institutional Animal Care and Use Committee, Docket # A-305, Animal Welfare Assurance Number A-3306-01.

### Mice

C57BL/6J (Ly5.2+) male mice were purchased from The Jackson Laboratory (Bar Harbor, ME). Ly5.1 and Thy1.1 P14 [73] TCR-transgenic mice were bred in the Department of Animal Medicine at the University of Massachusetts Medical School (UMMS). The P14 transgenic mice were bred onto the B6.IFNαβR KO background to generate P14 CD8 T cells that lacked IFNαβR [13], [74].

### Virus stocks and peptides

Lymphocytic choriomeningitis virus (LCMV), strain Armstrong, was propagated in baby hamster kidney (BHK21) cells, as previously described [75], [76]. Mice were injected intraperitoneally (i.p.) with 5×10^4^ pfu of LCMV. Organ homogenate viral titers were determined by plaque assay using Vero cells. To activate P14 CD8 T cells without a virus-induced inflammatory response, mice were inoculated intravenously (i.v.) with 5 µg (diluted in HBSS) of a 13-mer peptide (GP_33–45_) (KAVYNFATCGIFA) from the LCMV glycoprotein. RMA cells were labeled with the minimal GP33 epitope (KAVYNFATC), or the Vaccinia Virus K3L epitope (YSLPNAGDVI) at 1 µM concentration.

### Poly(I∶C) and cytokines

Poly(I∶C) was purchased from InvivoGen (SanDiego, CA) and diluted in HBSS for a concentration of 1 µg/µl. Mice were either inoculated with 200 µl HBSS or 200 µg poly(I∶C) i.p. Mouse IFNβ was purchased from PBL Interferon Source. Cytokines: IL-2 was purchased from BD Biosciences, IL-6 and IL-12 were purchased from R&D, and IL-7 and IL-15 were purchased from PeproTech, INC. Splenocytes were stimulated *ex vivo* with cytokine concentrations of 1000 U/ml IFNβ, or 10 µg/ml of IL-2, IL-6, IL-7, IL-12, or IL-15 at 37°C for ∼30 minutes.

### Adoptive transfers

For the dual transfer experiments, splenocytes (1–3×10^7^) from WT or IFNαβR KO P14 transgenic mice (Ly5.1+ or Thy1.1+) were adoptively transferred i.v. into congenic C57BL/6J (Ly5.2+ Thy1.2+) mice. One day after transfer, mice were inoculated i.p. with HBSS or poly(I∶C), and at various times post treatment (1 day∼18–24 hours, 2 days∼40–48 hours, 3 days∼64–72 hours) spleens were isolated, and the frequency of transgenic P14 CD8 T cells was determined by flow cytometric staining of Vα2, CD8α, and Ly5.1 or Thy1.1. Equal numbers (∼10.000) of P14 CD8 T cells were transferred i.v. into congenic B6 hosts immediately prior to infection with LCMV. For single transfer experiments, Ly5.1 or Thy1.1 P14 TCR transgenic mice were inoculated with HBSS or poly(I∶C) i.p. for various times, after which, the same protocol was used as the dual transfer method to transfer in equal numbers of P14 transgenic T cells. In experiments where control- and poly(I∶C)-treated transgenic P14 cells were transferred into the same recipients subsequently infected with LCMV, a total of 10,000 P14 cells were transferred. In experiments where HBSS- and poly(I∶C)- treated P14 cells were transferred into the same recipients receiving 13-mer GP_33–45_ peptide, ∼2–4×10^5^ P14 cells were transferred i.v. This higher amount was necessary for the peptide-stimulated cells to be detected. Prior to any adoptive transfer, single cell suspensions were prepared by lysing red blood cells with 0.84% NH_4_Cl solution and washing with HBSS.

Where described, cells were labeled with 5 µM CellTrace Violet (Invitrogen) by incubating at 37°C for 15 min. Cells were then washed with HBSS at least 2 times prior to adoptive transfer. A larger number of transgenic P14 cells (∼1×10^6^ per group) were transferred into hosts to identify virus-specific cells early after LCMV infection.

### Surface and intracellular cytokine staining

Spleen leukocytes were stained with a combination of fluorescently labeled monoclonal antibodies (MAb) specific for CD8α (53-6.7), Vα2 TCR (B20.1), Ly5.1 (A20), Thy1.1 (HIS51), CD44 (IM7) KLRG1 (2F1), CD127 (A7R34), and IFNAR1 (MAR1-5A3) for 20 min at 4°C. Intracellular cytokine staining was performed as described previously [45]. Briefly, spleen leukocytes (2–4×10^6^) were plated with or without 5 µM synthetic peptide stimulation in the presence of GolgiPlug (BD Pharmingen) and human rIL-2 for 4 to 5 hours at 37°C. After stimulation, cells were washed in Flow Cytometry Buffer (2% FCS in HBSS), blocked with α-FcR (2.4G2) and stained with a combination of fluorescently labeled mAbs listed above. After surface staining, spleen leukocytes were fixed and permeabilized with Cytofix/Cytoperm (BD Bioscience) for 20 min at 4°C and then stained with a combination of fluorescently labeled MAb specific for TNF (MP6-XT22), IFNγ (XMG1.2), and Granzyme B (GB11, Invitrogen). To identify cells undergoing Ag-induced degranulation, splenocytes were stimulated as stated above with addition of CD107a (1D4B) and CD107b (ABL-93). All MAbs were purchased from eBioscience, SanDiego, CA, BioLegend, San Diego, CA, or BD Bioscience, San Diego, CA. unless otherwise noted.

Freshly stained and previously fixed samples were acquired using a BD Bioscience LSR II flow cytometer with FACS Diva software. Data were analyzed with FlowJo software (Tree Star Inc., Ashland, OR). To determine percent divided and proliferation index, the proliferation function from FlowJo was applied to samples.

### Phosflow and intracellular protein staining

To identify intracellular proteins (phospho-specific STATs, total STAT levels, SOCS1, and BCL3) the BD Phosflow Alternative Protocol 1 was used and slightly modified. Generally, spleen leukocytes were isolated, stimulated (where appropriate), fixed, stained for surface molecules, permeabilized, and finally stained for intracellular proteins. Spleens were isolated, and single cell suspensions were prepared. Red blood cells were lysed by addition of 0.84% NH_4_Cl solution, and cells were plated at 2–4×10^6^ cells per well in 96 well round bottom plates. Cells were incubated at 37°C in 100 µl of media (RPMI supplemented with 10% FCS and pen-strep and L-Glut) for the indicated times in the presence (stimulated) or absence (unstimulated) of cytokines. Total volume was brought up to 200 µl before spinning. Cells were fixed with BD cytofix (BD Bioscience) on ice for 20 min, washed with Flow Cytometry Buffer and blocked with α-FcR (2.4G2) for 5 min at 4°C. Cells were washed and stained with a variety of fluorescently labeled MAbs for 20 min at 4°C, washed with Flow Cytometry Buffer, and then permeablized with BD Perm buffer III (BD Bioscience) for 30 min on ice. Splenocytes were washed and then stained with a combination of fluorescently labeled Abs pY701 STAT1 (BD Bioscience), pY705 STAT3 (BD Bioscience), pY693 STAT4 (BD Bioscience), pY694 STAT5 (BD Bioscience), STAT1 (clone 1/Stat1; BD Bioscience), or unlabeled Abs STAT3 (79D7; cell signaling technology), STAT4 (C46B10; cell signaling technology), STAT5 (3H7; cell signaling technology), SOCS1 (A156; cell signaling technology), or BCL3 (C-14; Santa Cruz Biotechnology) for 20–30 min at RT in the dark. If antibodies were not fluorescently labeled, cells were washed and then stained with FITC-labeled donkey anti-Rabbit IgG (Poly4064; BioLegend) for 15 min at RT in the dark. After intracellular staining, splenocytes were washed and samples were acquired using a BD Bioscience LSR II flow cytometry with FACS Diva software. Data were analyzed with FlowJo software.

### RNA isolation and quantitative real time PCR

Naïve CD44lo CD8 T cells were sorted to 98–99% purity using MACS Naïve CD8a+ T cell Isolation Kit (Miltenyi Biotec). RNA was isolated from sorted naïve CD8 T cells with an RNeasy mini kit (Qiagen) and concentration was determined. cDNA was generated using the RT^2^ Easy First Strand Kit (Qiagen) and QuantiFast SYBR Green PCR Kit (Qiagen) was used to determine the relative mRNA concentrations by quantitative real-time PCR. Primers for *Socs1* (RefSeq Accession number NM_009896.2) and *Actb* (RefSeq Accession number NM_007393.3) were used.

### Trogocytosis assay

When CD8 T cells kill target cells, they strip off part of the target cell membrane and incorporate it into their own, by a process called trogocytosis [53], [54]. A trogocytosis assay was thus performed to measure the ability of P14 T cells to attack targets. Effector P14 cells were generated as described in earlier materials and methods sections. Briefly, P14 mice were either HBSS or poly(I∶C) treated for 1 day prior to adoptive transfer ∼10,000 total P14 cells per group into separate animals subsequently infected with LCMV. Spleens were harvested at day 5 post infection and single cell suspensions were obtained. Targets were RMA cells cultured in complete RPMI and were un-pulsed, pulsed with an irrelevant peptide, K3L (YSLPNAGDVI) or pulsed with a specific peptide, GP33 (KAVYNFATC), at 1 µM for ∼90–120 minutes at 37°C. After incubation, target cells were labeled with fluorescent lipids SP-DiIC_18_(3) (Molecular probes) and diluted in Diluent C (Sigma Aldrich) using the protocol adapted from Daubeuf S. et al [54]. Target cells (∼7×10^5^) were co-cultured with effector cells (1.5×10^6^ total splenocytes) per well for 1 hour at 37°C. Cells were stained with surface antibodies of interest, and samples were acquired using a BD Bioscience LSR II flow cytometry with FACS Diva software. Data were analyzed with FlowJo software.

### Statistical analyses

Where appropriate, Students t test and linear regression were calculated using GraphPad InStat software. Significance was set at a P value of 0.05; * indicates a P of <0.05, ** a P of <0.01, *** a P of <0.001, and **** a P of <0.0001. All results are expressed as means of +/− standard deviations.

## Supporting Information

Figure S1 **Poly(I∶C)-pretreated memory phenotype CD8 T cells are refractory to IFNβ-induced STAT phosphorylation.** As described in **Figure 2** and Materials and Methods, mice were HBSS (open bars) or poly(I∶C) (black bars) treated for 1 day. Splenocytes were isolated and were either unstimulated, or stimulated *ex vivo* with IFNβ for 30 min and then stained for (A) pSTAT1, (B) pSTAT3, (C), pSTAT4, and (D) pSTAT5. Cells were gated on CD44hi CD8α+ lymphocytes, and the MFI of each respective pSTAT is graphed. Data are representative of at least 4 independent experiments with n of 3 mice per group. Statistical analysis is described in Materials and Methods section.
(EPS)

Figure S2 **Naïve CD44lo CD8 T cells do not phosphorylate downstream STATs in response to other cytokines.** As described in **Figure 4** and Materials and Methods, mice were HBSS (open bars) or poly(I∶C) (black bars) treated for 1 day. Splenocytes were isolated and were unstimulated, stimulated with IFNβ, IL-2, IL-7, or IL-12 for 30 min and stained for appropriate downstream STAT molecules (A–B) pSTAT5 MFI, and (C) pSTAT4 MFI. Splenocytes were gated on CD44lo CD8α+ lymphocytes. (A) responsiveness to IFNβ and IL-2, (B) responsiveness to IFNβ and IL-7, and (C) responsiveness to IFNβ and IL-12. Data are representative of at least 2 independent experiments with n of 3 mice per group.
(EPS)

Figure S3 **CD44hi CD8 T cells respond to some cytokines after 1 day of poly(I∶C) treatment.** As described in **Figure 4**, mice were inoculated with HBSS(open bars) or poly(I∶C) (black bars) for 1 day. Splenocytes were isolated and either unstimulated or stimulated with IL-6 (A), or IL-15 (B) and stained for downstream pSTAT3 (A) or pSTAT5 (B). Splenocytes were gated on CD44hi CD8α+ lymphocytes and plotted for pSTAT MFI. Data are representative of at least 2 independent experiments with n of 3 mice per group.
(EPS)

Figure S4 **Cytokine receptor expression after 1, 2, or 3 days of poly(I∶C)-pretreatment.** As described in **Figure 5**, mice were given one dose of HBSS (open bars) or poly(I∶C) (black bars) and harvested at 1, 2, or 3 days after the inoculation. Cytokine receptor expression was determined on the CD44lo CD8α+ T cells. The MFI is plotted for (A) CD25, (B) CD122, (C) CD126, (D) CD127, and (E) CD132. Cytokine receptors tested include IL-2 (A–B, E), IL-6 (C), IL-7 (D–E) and IL-15 (B, E). Data are representative of 2 independent experiments with n of 3 mice per group.
(EPS)

Figure S5 **Memory phenotype CD8 T cells increase SOCS1 expression after poly(I∶C) treatment.** As described in **Figure 5**, mice were given one dose of HBSS (open bars) or poly(I∶C) (black bars) and harvested at 1, 2, or 3 days after the inoculation. Splenocytes were gated on CD44hi CD8α+ T cells showing MFI of (A) IFNAR1 and (B) SOCS1. Data are representative of 2 independent experiments with n of 3 mice per group.
(EPS)

Figure S6 **Trogocytosis capability of HBSS- and poly(I∶C)-pretreated P14 cells co-cultured with GP33 pulsed RMA cells.** As described in the Materials and Methods section, a trogocytosis assay was performed using day 5 HBSS- or poly(I∶C)-pretreated P14 CD8 T cells as effectors and RMA cells pulsed with peptides as targets. Effectors were developed by poly(I∶C) or HBSS treating a P14 transgenic mouse, transferring ∼10,000 P14 cells from each group into separate mice 1 day after treatment and infecting the recipient mice with LCMV. At day 5 post infection, splenocytes containing the donor P14 CD8 T cells were isolated and used as effectors. Target cells were RMA cells that were not pulsed with peptide (no peptide), pulsed with an irrelevant peptide (K3L), or pulsed with the specific peptide (GP33). Target cells were labeled with fluorescent lipid molecule SP-DiIC_18_(3) that can be detected if it is transferred to a different cell through trogocytosis. Target cells were in excess and were co-incubated with effectors for 1 hour, stained with surface antibodies and ran on a flow cytometer. (A) shows representative FACS plots gated on donor P14 cells that were HBSS or poly(I∶C) pretreated co-incubated with 1. No targets, 2. No peptide pulsed targets, 3. K3L pulsed targets, or 4. GP33 pulsed targets, looking at P14 cell incorporation of SP-DiIC_18_(3). Data are representative of 2 independent experiments with n of 3–5 mice per group. (B) MFI of SP-DiIC_18_(3) gated on donor P14 cells, normalized to HBSS control for K3L and GP33 pulsed targets. HBSS pretreated P14 cells are in the open bars and poly(I∶C)-pretreated P14 cells represented as black bars. Data are combined from 2 independent experiments with a total n of 8 mice per group.
(EPS)

## References

[1] von Pirquet C (1908) Das verhalten der kutanen tuberkulin-reaktion wahrend der Masern. Dtsch Med Wochenschr 34: 1297–1300.

[2] Meyaard L (1992) Programmed death of T cells in HIV-1 infection. Science (New York, NY) 257: 217–219. 10.1126/science.1352911 1352911

[3] Mims CA, Wainwright S (1968) The Immunodepressive Action of Lymphocytic Choriomeningitis Virus in Mice. J Immunol 101: 717–724. 5681651

[4] Osborn JE, Blazkovec AA, Walker DL (1968) Immunosuppression during Acute Murine Cytomegalovirus Infection. J Immunol 100: 835–844. 4296301

[5] Kantzler GB, Lauteria SF, Cusumano CL, Lee JD, Ganguly R, et al (1974) Immunosuppression During Influenza Virus Infection. Infect Immun 10: 996–1002. 16558116 10.1128/iai.10.5.996-1002.1974 PMC423051

[6] Welsh RM, Selin LK, Razvi ES (1995) Role of apoptosis in the regulation of virus-induced T cell responses, immune suppression, and memory. J Cell Biochem 59: 135–142. 8904307 10.1002/jcb.240590202

[7] Rouse BT, Horohov DW (1986) Immunosuppression in Viral Infections. Rev Infect Dis 8: 850–873. 3025993 10.1093/clinids/8.6.850 PMC7792945

[8] Zarozinski CC, McNally JM, Lohman BL, Daniels KA, Welsh RM (2000) Bystander Sensitization to Activation-Induced Cell Death as a Mechanism of Virus-Induced Immune Suppression. J Virol 74: 3650–3658. 10729141 10.1128/jvi.74.8.3650-3658.2000 PMC111875

[9] Razvi ES, Welsh RM (1993) Programmed cell death of T lymphocytes during acute viral infection: a mechanism for virus-induced immune deficiency. J Virol 67: 5754–5765. 8371341 10.1128/jvi.67.10.5754-5765.1993 PMC237993

[10] Mathew A, Kurane I, Green S, Vaughn DW, Kalayanarooj S, et al (1999) Impaired T Cell Proliferation in Acute Dengue Infection. J Immunol 162: 5609–5615. 10228044

[11] Zuniga EI, Liou L-Y, Mack L, Mendoza M, Oldstone MBA (2008) Persistent Virus Infection Inhibits Type I Interferon Production by Plasmacytoid Dendritic Cells to Facilitate Opportunistic Infections. Cell Host Microbe 4: 374–386. 18854241 10.1016/j.chom.2008.08.016 PMC2875928

[12] Díaz-San Segundo F, Rodríguez-Calvo T, de Avila A, Sevilla N (2009) Immunosuppression during Acute Infection with Foot-and-Mouth Disease Virus in Swine Is Mediated by IL-10. PLoS ONE 4: e5659. 19478852 10.1371/journal.pone.0005659 PMC2682558

[13] Marshall HD, Urban SL, Welsh RM (2011) Virus-Induced Transient Immune Suppression and the Inhibition of T Cell Proliferation by Type I Interferon. J Virol 85: 5929–5939. 21471240 10.1128/JVI.02516-10 PMC3126308

[14] Mescher MF, Curtsinger JM, Agarwal P, Casey KA, Gerner M, et al (2006) Signals required for programming effector and memory development by CD8+ T cells. Immunol Rev 211: 81–92. 16824119 10.1111/j.0105-2896.2006.00382.x

[15] Curtsinger JM, Mescher MF (2010) Inflammatory cytokines as a third signal for T cell activation. Curr Opin Immunol 22: 333–340. 20363604 10.1016/j.coi.2010.02.013 PMC2891062

[16] Curtsinger JM, Johnson CM, Mescher MF (2003) CD8 T Cell Clonal Expansion and Development of Effector Function Require Prolonged Exposure to Antigen, Costimulation, and Signal 3 Cytokine. J Immunol 171: 5165–5171. 14607916 10.4049/jimmunol.171.10.5165

[17] Obar JJ, Lefrançois L (2010) Early events governing memory CD8+ T-cell differentiation. Int Immunol 22: 619–625. 20504887 10.1093/intimm/dxq053 PMC2908475

[18] Xiao Z, Casey KA, Jameson SC, Curtsinger JM, Mescher MF (2009) Programming for CD8 T Cell Memory Development Requires IL-12 or Type I IFN. J Immunol 182: 2786–2794. 19234173 10.4049/jimmunol.0803484 PMC2648124

[19] Sikora AG, Jaffarzad N, Hailemichael Y, Gelbard A, Stonier SW, et al (2009) IFN-α Enhances Peptide Vaccine-Induced CD8+ T Cell Numbers, Effector Function, and Antitumor Activity. J Immunol 182: 7398–7407. 19494262 10.4049/jimmunol.0802982 PMC2774140

[20] Curtsinger JM, Lins DC, Mescher MF (2003) Signal 3 Determines Tolerance versus Full Activation of Naive CD8 T Cells. J Exp Med 197: 1141–1151. 12732656 10.1084/jem.20021910 PMC2193970

[21] Curtsinger JM, Valenzuela JO, Agarwal P, Lins D, Mescher MF (2005) Cutting Edge: Type I IFNs Provide a Third Signal to CD8 T Cells to Stimulate Clonal Expansion and Differentiation. J Immunol 174: 4465–4469. 15814665 10.4049/jimmunol.174.8.4465

[22] Agarwal P, Raghavan A, Nandiwada SL, Curtsinger JM, Bohjanen PR, et al (2009) Gene Regulation and Chromatin Remodeling by IL-12 and Type I IFN in Programming for CD8 T Cell Effector Function and Memory. J Immunol 183: 1695–1704. 19592655 10.4049/jimmunol.0900592 PMC2893405

[23] Thompson LJ, Kolumam GA, Thomas S, Murali-Krishna K (2006) Innate Inflammatory Signals Induced by Various Pathogens Differentially Dictate the IFN-I Dependence of CD8 T Cells for Clonal Expansion and Memory Formation. J Immunol 177: 1746–1754. 16849484 10.4049/jimmunol.177.3.1746

[24] Keppler SJ, Aichele P (2011) Signal 3 requirement for memory CD8+ T-cell activation is determined by the infectious pathogen. Eur J Immunol 41: 3176–3186. 21830209 10.1002/eji.201141537

[25] Keppler SJ, Rosenits K, Koegl T, Vucikuja S, Aichele P (2012) Signal 3 Cytokines as Modulators of Primary Immune Responses during Infections: The Interplay of Type I IFN and IL-12 in CD8 T Cell Responses. PLoS ONE 7: e40865. 22815848 10.1371/journal.pone.0040865 PMC3398954

[26] Pham N-L, Badovinac V, Harty J (2011) Differential role of “signal 3” inflammatory cytokines in regulating CD8 T cell expansion and differentiation in vivo. Front Immunol 2: 4. 22566795 10.3389/fimmu.2011.00004 PMC3342074

[27] Kolumam GA, Thomas S, Thompson LJ, Sprent J, Murali-Krishna K (2005) Type I interferons act directly on CD8 T cells to allow clonal expansion and memory formation in response to viral infection. J Exp Med 202: 637–650. 16129706 10.1084/jem.20050821 PMC2212878

[28] Aichele P, Unsoeld H, Koschella M, Schweier O, Kalinke U, et al (2006) Cutting Edge: CD8 T Cells Specific for Lymphocytic Choriomeningitis Virus Require Type I IFN Receptor for Clonal Expansion. The Journal of Immunology 176: 4525–4529. 16585541 10.4049/jimmunol.176.8.4525

[29] Keppler SJ, Theil K, Vucikuja S, Aichele P (2009) Effector T-cell differentiation during viral and bacterial infections: Role of direct IL-12 signals for cell fate decision of CD8+ T cells. Eur J Immunol 39: 1774–1783. 19548244 10.1002/eji.200839093

[30] Obar JJ, Jellison ER, Sheridan BS, Blair DA, Pham Q-M, et al (2011) Pathogen-Induced Inflammatory Environment Controls Effector and Memory CD8+ T Cell Differentiation. J Immunol 187: 4967–4978. 21987662 10.4049/jimmunol.1102335 PMC3208080

[31] Mitchell TC, Hildeman D, Kedl RM, Teague TK, Schaefer BC, et al (2001) Immunological adjuvants promote activated T cell survival via induction of Bcl-3. Nat Immunol 2: 397–402. 11323692 10.1038/87692

[32] Mitchell TC, Teague TK, Hildeman DA, Bender J, Rees WA, et al (2002) Stronger Correlation of bcl-3 than bcl-2, bcl-xL, Costimulation, or Antioxidants with Adjuvant-Induced T Cell Survival. Ann N Y Acad Sci 975: 114–131. 12538159 10.1111/j.1749-6632.2002.tb05946.x

[33] Valenzuela JO, Hammerbeck CD, Mescher MF (2005) Cutting Edge: Bcl-3 Up-Regulation by Signal 3 Cytokine (IL-12) Prolongs Survival of Antigen-Activated CD8 T Cells. J Immunol 174: 600–604. 15634875 10.4049/jimmunol.174.2.600

[34] Joshi NS, Cui W, Chandele A, Lee HK, Urso DR, et al (2007) Inflammation Directs Memory Precursor and Short-Lived Effector CD8+ T Cell Fates via the Graded Expression of T-bet Transcription Factor. Immunity 27: 281–295. 17723218 10.1016/j.immuni.2007.07.010 PMC2034442

[35] Wiesel M, Crouse J, Bedenikovic G, Sutherland A, Joller N, et al (2012) Type-I IFN drives the differentiation of short-lived effector CD8+ T cells in vivo. Eur J Immunol 42: 320–329. 22102057 10.1002/eji.201142091

[36] Curtsinger JM, Lins DC, Johnson CM, Mescher MF (2005) Signal 3 Tolerant CD8 T Cells Degranulate in Response to Antigen but Lack Granzyme B to Mediate Cytolysis. J Immunol 175: 4392–4399. 16177080 10.4049/jimmunol.175.7.4392

[37] Hervas-Stubbs S, Perez-Gracia JL, Rouzaut A, Sanmamed MF, Le Bon A, et al (2011) Direct Effects of Type I Interferons on Cells of the Immune System. Clin Cancer Res 17: 2619–2627. 21372217 10.1158/1078-0432.CCR-10-1114

[38] Gil MP, Salomon R, Louten J, Biron CA (2006) Modulation of STAT1 protein levels: a mechanism shaping CD8 T-cell responses in vivo. Blood 107: 987–993. 16210337 10.1182/blood-2005-07-2834 PMC1895900

[39] Nguyen KB, Cousens LP, Doughty LA, Pien GC, Durbin JE, et al (2000) Interferon [alpha]/[beta]-mediated inhibition and promotion of interferon [gamma]: STAT1 resolves a paradox. Nat Immunol 1: 70–76. 10881178 10.1038/76940

[40] Gimeno R, Lee C-K, Schindler C, Levy DE (2005) Stat1 and Stat2 but Not Stat3 Arbitrate Contradictory Growth Signals Elicited by Alpha/Beta Interferon in T Lymphocytes. Mol Cell Biol 25: 5456–5465. 15964802 10.1128/MCB.25.13.5456-5465.2005 PMC1156979

[41] Tanabe Y, Nishibori T, Su L, Arduini RM, Baker DP, et al (2005) Cutting Edge: Role of STAT1, STAT3, and STAT5 in IFN-αβ Responses in T Lymphocytes. J Immunol 174: 609–613. 15634877 10.4049/jimmunol.174.2.609

[42] Nguyen KB, Watford WT, Salomon R, Hofmann SR, Pien GC, et al (2002) Critical Role for STAT4 Activation by Type 1 Interferons in the Interferon-γ Response to Viral Infection. Science 297: 2063–2066. 12242445 10.1126/science.1074900

[43] Gil MP, Ploquin MJY, Watford WT, Lee S-H, Kim K, et al (2012) Regulating type 1 IFN effects in CD8 T cells during viral infections: changing STAT4 and STAT1 expression for function. Blood 120: 3718–3728. 22968462 10.1182/blood-2012-05-428672 PMC3488885

[44] Welsh RM, Bahl K, Marshall HD, Urban SL (2012) Type 1 Interferons and Antiviral CD8 T-Cell Responses. PLoS Pathog 8: e1002352. 22241987 10.1371/journal.ppat.1002352 PMC3252364

[45] Marshall HD, Prince AL, Berg LJ, Welsh RM (2010) IFN-αβ and Self-MHC Divert CD8 T Cells into a Distinct Differentiation Pathway Characterized by Rapid Acquisition of Effector Functions. J Immunol 185: 1419–1428. 20592282 10.4049/jimmunol.1001140 PMC3232037

[46] Cheon H, Yang J, George R Stark (2011) The Functions of Signal Transducers and Activators of Transcriptions 1 and 3 as Cytokine-Inducible Proteins. J Interferon Cytokine Res 31: 33–40. 21166594 10.1089/jir.2010.0100 PMC3021352

[47] Yoshimura A, Suzuki M, Sakaguchi R, Hanada T, Yasukawa H (2012) SOCS, inflammation and autoimmunity. Front Immunol 3: 20. 22566904 10.3389/fimmu.2012.00020 PMC3342034

[48] Aaronson DS, Horvath CM (2002) A Road Map for Those Who Don't Know JAK-STAT. Science 296: 1653–1655. 12040185 10.1126/science.1071545

[49] Piganis RAR, De Weerd NA, Gould JA, Schindler CW, Mansell A, et al (2011) Suppressor of Cytokine Signaling (SOCS) 1 Inhibits Type I Interferon (IFN) Signaling via the Interferon α Receptor (IFNAR1)-associated Tyrosine Kinase Tyk2. J Biol Chem 286: 33811–33818. 21757742 10.1074/jbc.M111.270207 PMC3190811

[50] Palmer DC, Restifo NP Suppressors of cytokine signaling (SOCS) in T cell differentiation, maturation, and function. Trends Immunol 30: 592–602. 19879803 10.1016/j.it.2009.09.009 PMC2787651

[51] Zheng H, Qian J, Varghese B, Baker DP, Fuchs S (2011) Ligand-Stimulated Downregulation of the Alpha Interferon Receptor: Role of Protein Kinase D2. Mol Cell Biol 31: 710–720. 21173164 10.1128/MCB.01154-10 PMC3028644

[52] Kaech SM, Tan Joyce T, Wherry John E, Konieczny Bogumila T, Surh Charles D, Ahmed Rafi (2003) Selective expression of the interleukin 7 receptor identifies effector CD8 T cells that give rise to long-lived memory cells. Nat Immunol 4: 1191–1198. 14625547 10.1038/ni1009

[53] Puaux A-L, Campanaud J, Salles A, Préville X, Timmerman B, et al (2006) A very rapid and simple assay based on trogocytosis to detect and measure specific T and B cell reactivity by flow cytometry. Eur J Immunol 36: 779–788. 16482513 10.1002/eji.200535407

[54] Daubeuf S (2006) A simple trogocytosis-based method to detect, quantify, characterize and purify antigen-specific live lymphocytes by flow cytometry, via their capture of membrane fragments from antigen-presenting cells. Nat Protoc 1: 2536–2542. 17406507 10.1038/nprot.2006.400

[55] Hudrisier D, Riond J, Mazarguil H, Gairin JE, Joly E (2001) Cutting Edge: CTLs Rapidly Capture Membrane Fragments from Target Cells in a TCR Signaling-Dependent Manner. J Immunol 166: 3645–3649. 11238601 10.4049/jimmunol.166.6.3645

[56] Hudrisier D, Riond J, Garidou L, Duthoit C, Joly E (2005) T cell activation correlates with an increasedproportion of antigen among the materials acquiredfrom target cells. Eur J Immunol 35: 2284–2294. 16021601 10.1002/eji.200526266

[57] Akbar AN, Borthwick N, Salmon M, Gombert W, Bofill M, et al (1993) The significance of low bcl-2 expression by CD45RO T cells in normal individuals and patients with acute viral infections. The role of apoptosis in T cell memory. J Exp Med 178: 427–438. 8340752 10.1084/jem.178.2.427 PMC2191107

[58] Moutaftsi M, Mehl AM, Borysiewicz LK, Tabi Z (2002) Human cytomegalovirus inhibits maturation and impairs function of monocyte-derived dendritic cells. Blood 99: 2913–2921. 11929782 10.1182/blood.v99.8.2913

[59] Welsh RM (2001) Immunology. Brief encounter. Nature (London) 411: 541–542. 11385554 10.1038/35079229

[60] van Stipdonk MJB, Lemmens EE, Schoenberger SP (2001) Naive CTLs require a single brief period of antigenic stimulation for clonal expansion and differentiation. Nat Immunol 2: 423–429. 11323696 10.1038/87730

[61] Kaech SM, Ahmed R (2001) Memory CD8+ T cell differentiation: initial antigen encounter triggers a developmental program in naive cells. Nat Immunol 2: 415–422. 11323695 10.1038/87720 PMC3760150

[62] Mercado R, Vijh S, Allen SE, Kerksiek K, Pilip IM, et al (2000) Early Programming of T Cell Populations Responding to Bacterial Infection. J Immunol 165: 6833–6839. 11120806 10.4049/jimmunol.165.12.6833

[63] Wiedmann M, Liebert UG, Oesen U, Porst H, Wiese M, et al (2000) Decreased immunogenicity of recombinant hepatitis B vaccine in chronic hepatitis C. Hepatology 31: 230–234. 10613751 10.1002/hep.510310134

[64] Laurence JC (2005) Hepatitis A and B immunizations of individuals infected with human immunodeficiency virus. Am J Med 118: 75–83. 10.1016/j.amjmed.2005.07.024 16271546

[65] Leroy V (2002) The antibody response to hepatitis B virus vaccination is negatively influenced by the hepatitis C virus viral load in patients with chronic hepatitis C: a case-control study. Eur J Gastroenterol Hepatol 14: 485–489. 11984145 10.1097/00042737-200205000-00004

[66] Abu-Shakra M, Press J, Varsano N, Levy V, Mendelson E, et al (2002) Specific antibody response after influenza immunization in systemic lupus erythematosus. J Rheumatol 29: 2555–2557. 12465151

[67] Holvast A, van Assen S, de Haan A, Huckriede A, Benne CA, et al (2009) Studies of cell-mediated immune responses to influenza vaccination in systemic lupus erythematosus. Arthritis Rheum 60: 2438–2447. 19644961 10.1002/art.24679

[68] Welsh RM (1984) Natural killer cells and interferon. Crit Rev Immunol 5: 55–93. 6085941

[69] Le Bon A, Thompson C, Kamphuis E, Durand V, Rossmann C, et al (2006) Cutting Edge: Enhancement of Antibody Responses Through Direct Stimulation of B and T Cells by Type I IFN. J Immunol 176: 2074–2078. 16455962 10.4049/jimmunol.176.4.2074

[70] Welsh JR (1978) Cytotoxic cells induced during lymphocytic choriomeningitis virus infection of mice. I. Characterization of natural killer cell induction. J Exp Med 148: 163–181. 307587 10.1084/jem.148.1.163 PMC2184910

[71] Schrier RD, Rice GPA, Oldstone MBA (1986) Suppression of Natural Killer Cell Activity and T Cell Proliferation by Fresh Isolates of Human Cytomegalovirus. J Infect Dis 153: 1084–1091. 2422296 10.1093/infdis/153.6.1084

[72] Mitchell TC, Thompson BS, Trent JO, Casella CR (2002) A Short Domain within Bcl-3 Is Responsible for Its Lymphocyte Survival Activity. Ann N Y Acad Sci 975: 132–147. 12538160 10.1111/j.1749-6632.2002.tb05947.x

[73] Pircher H, Burki Kurt, Lang Rosemarie, Hengartner Hans, Zinkernagel Rolf M (1989) Tolerance induction in double specific T-cell receptor transgenic mice varies with antigen. Nature 342: 559–561. 2573841 10.1038/342559a0

[74] Muller U, Steinhoff Ulrich, Reis Luis FL, Hemmi Silvio, Pavlovic Jovan, Zinkernagel Rolf M (1994) Functional role of Type I and Type II interferons in antiviral defense. Science 264: 1918–1921. 8009221 10.1126/science.8009221

[75] Welsh RM, Seedhom MO (2008) Lymphocytic Choriomeningitis Virus (LCMV): Propagation, Quantitation, and Storage. Curr Protoc Microbiol John Wiley & Sons, Inc. 10.1002/9780471729259.mc15a01s8 PMC3220596 18770534

[76] Yang HY, Dundon PL, Nahill SR, Welsh RM (1989) Virus-induced polyclonal cytotoxic T lymphocyte stimulation. J Immunol 142: 1710–1718. 2537363

